# Colonizing Microbes, IL-10 and IL-22: Keeping the Peace at the Mucosal Surface

**DOI:** 10.3389/fmicb.2021.729053

**Published:** 2021-09-17

**Authors:** Evelien Kidess, Michiel Kleerebezem, Sylvia Brugman

**Affiliations:** Animal Sciences Group, Host-Microbe Interactomics, Wageningen University and Research, Wageningen, Netherlands

**Keywords:** IL-10, IL-22, zebrafish, microbiota, mice, epithelial homeostasis, intestines

## Abstract

Our world is filled with microbes. Each multicellular organism has developed ways to interact with this microbial environment. Microbes do not always pose a threat; they can contribute to many processes that benefit the host. Upon colonization both host and microbes adapt resulting in dynamic ecosystems in different host niches. Regulatory processes develop within the host to prevent overt inflammation to beneficial microbes, yet keeping the possibility to respond when pathogens attempt to adhere and invade tissues. This review will focus on microbial colonization and the early (innate) host immune response, with special emphasis on the microbiota-modifying roles of IL-10 and IL-22 in the intestine. IL-10 knock out mice show an altered microbial composition, and spontaneously develop enterocolitis over time. IL-22 knock out mice, although not developing enterocolitis spontaneously, also have an altered microbial composition and increase of epithelial-adherent bacteria, mainly caused by a decrease in mucin and anti-microbial peptide production. Recently interesting links have been found between the IL-10 and IL-22 pathways. While IL-22 can function as a regulatory cytokine at the mucosal surface, it also has inflammatory roles depending on the context. For example, lack of IL-22 in the IL-10–/– mice model prevents spontaneous colitis development. Additionally, the reduced microbial diversity observed in IL-10–/– mice was also reversed in IL-10/IL-22 double mutant mice (Gunasekera et al., 2020). Since in early life, host immunity develops in parallel and in interaction with colonizing microbes, there is a need for future studies that focus on the effect of the timing of colonization in relation to the developmental phase of the host. To illustrate this, examples from zebrafish research will be compared with studies performed in mammals. Since zebrafish develop from eggs and are directly exposed to the outside microbial world, timing of the development of host immunity and subsequent control of microbial composition, is different from mammals that develop *in utero* and only get exposed after birth. Likewise, colonization studies using adult germfree mice might yield different results from those using neonatal germfree mice. Lastly, special emphasis will be given to the need for host genotype and environmental (co-housing) control of experiments.

## Microbial Colonization

As soon as organisms come into contact with the outside world the process of colonization starts. In some animals colonization starts at birth, while in others (such as fish) colonization of the eggs immediately starts when the eggs of the female are released into the surrounding water (Egerton et al., 2018). At 3 days post fertilization (dpf) the mouth of the fish opens and microbes can colonize the developing gastrointestinal tract (Kimmel et al., 1995). From the microbial perspective, colonization of a niche is often driven by the availability of substrates that allows growth and population expansion. As with any microbial colonization process of a new niche, it is initiated by pioneers, which are followed or sometimes replaced by other groups of organisms. The microbial ecosystem of host-associated niches commonly becomes more complex during these secondary stages, which are often including the colonization by species that can utilize the metabolic products of the pioneers as substrate for growth, thereby, establishing syntrophic chains and assembling an interactive microbial food-web (Sung et al., 2017; D’Hoe et al., 2018). Competition and cooperative processes will simultaneously lead to a more or less stable microbial community which we call microbiota.

Colonization of the gastrointestinal tract depends on several biotic (nutrients, other microbes, etc.) and abiotic (pH, temperature, etc.) factors. Furthermore, the onset of colonization can be different for different species. For example, colonization in mammals begins at birth (although there is some discussion on the sterility of the womb, Perez-Munoz et al., 2017), while in fish, spawned eggs directly acquire a microbiota (Liu et al., 2014). Therefore, the selective pressures on and later within the host, but also the (timing of the) development of immune responses toward microbes can be very different depending on the host species studied.

Recently, a large cohort study (TEDDY), typing 903 children (12,500 stool samples) from six different locations (European and United States), showed that during early life in humans, three distinct phases of microbial community structure could be discriminated: a developmental phase in roughly the first year, a transitional phase in the second year (<30 months), and a stable phase from ≥31 months (Stewart et al., 2018). Breastfeeding explained most microbial composition variance in the developmental phase, with those children receiving breastmilk showing a modestly higher abundance of *Bifidobacterium* (but also *Lactobacillus rhamnosus* and *Staphylococcus epidermidis*) compared to children that were formula fed that showed (modest) higher abundance of *Escherichia coli*. The metabolic genes enriched in the microbiome of breastfed children correlated with the presence of high concentrations on human milk oligosaccharides (HMOs) in breastmilk. The change into the transition phase seemed to be caused by the cessation of breastmilk rather than the start of introduction of solid food, confirming previous studies (Bergstrom et al., 2014; Backhed et al., 2015; Pannaraj et al., 2017). Infant formula is frequently supplemented with galacto- and/or fructo-oligosaccharides, which (in part) probably compensates for the difference in Bifidobacterial abundance during the earlier stages of life, explaining why smaller scaled studies may not have detected the association of this genus with breastfeeding (Timmerman et al., 2017).

The distinct phases of microbial community shifts were also reported in zebrafish. Longitudinal evaluation of the microbial composition in siblings from a single parent pair that were housed in separate tanks revealed that the microbiota followed a distinct developmental pattern (Stephens et al., 2016). In the early time points [4–10 days post fertilization (dpf)] the larval zebrafish microbiota was more similar to the environmental samples, and the diversity of the population was relatively high. Around 10 dpf this began to shift, resulting in two larval microbial subpopulations, one resembling the richness of the 4 dpf samples, the other group shifting more toward older samples. At this time point of 10 dpf, zebrafish should have begun feeding. This is a pivotal life-stage in the larvae, since failure to feed from 5 dpf onward results in starvation and death between 10 and 12 dpf (Samuel et al., 2019). It is likely that the ability to feed causes the bimodal distribution in the 10 dpf samples. Over time in the zebrafish the relative abundance of Proteobacteria decreases (although it remains the dominant phylum) and the relative abundance of Fusobacteria and Firmicutes increases (Rawls et al., 2004, 2006; Roeselers et al., 2011; Stephens et al., 2016). Furthermore, the richness and phylogenetic diversity decreases from larval to adult stage (Stephens et al., 2016). This microbiota data was also used in a microbial modeling study which revealed that especially during early developmental stages, passive migration and stochastic demographic processes play an important role in microbial succession and colonization (Burns et al., 2016).

Rearing zebrafish under different environmental conditions until 12 days post hatching (12 dph, ∼14 dpf) and subsequently changing the environment and sampling until 98 dph showed that ecological succession of gut microbial communities mainly associates with developmental stages rather than the hatching environment (Xiao et al., 2021). Additional research is required to decipher the influence of neutral versus non-neutral processes (selective pressures) that might influence different microbes within the microbial composition at different stages of life.

For the colonizing microbes, the intestine is a specific niche where they find food, but also experience selective pressures. Here, oxygen level, feed, and the developing immune system play important intertwined roles (Bevins and Salzman, 2011). In a meta-analysis, Sullam et al. (2012) investigated the gut microbiota of different fish species with different habitats and feeding behavior. Although differences exist between different environments, fish species and trophic levels, the fish gut microbiota still clustered with gut communities from other species (mammals and insects) and not with free-living environmental bacteria, showing that the gut environment is a remarkably consistent selective ecological niche (Sullam et al., 2012). Microbes also adapt to living within this specific niche. For example, *Bacteroides* has been shown to adapt by upregulating so-called commensal colonization factors (ccf) when in contact with the mouse colon, but not under laboratory culture conditions (Lee et al., 2013). In another experiment, *Aeromonas veronii* was used as a model species and ‘passaged’ through populations of germfree larval zebrafish (Robinson et al., 2018). Each time the gut-associated *Aeromonas* population was inoculated in the aquatic environment of the next zebrafish population. Here, the authors showed that early adaptations in the microbe seemed to enhance initial colonization, while later adaptations were involved in host specialization (Robinson et al., 2018).

## Host–Microbe Interactions

Next to adaptation by the colonizing microbes, the host also responds to these new colonizers. The host needs to launch adequate defense reactions to counteract microbes that may cause damage to its mucosal surfaces, but at the same time it needs to be permissive to those bacteria that do not cause harm, or even support the host’s nutrient digestion processes. The host response can be well monitored by the exposure of germfree animals to conventional animal housing, enabling a new microbiota to be established. This process is also termed conventionalization. A large part of the host genes induced by conventionalization are immune-related and metabolic genes (El Aidy et al., 2012, 2013a,c; Hooper et al., 2012; Belkaid and Naik, 2013). Comparing conventionalization of zebrafish and mice showed that this response toward the microbiota is partly conserved between different host species (Rawls et al., 2004). Colonizing 3 dpf germfree zebrafish with bacteria from conventional zebrafish resulted in a transcriptional response of 212 genes (Rawls et al., 2004). Remarkably, but perhaps not surprisingly given the largely conserved pattern recognition receptors reacting to microbes in zebrafish and mammals (Li et al., 2017), 59 of these microbial colonization response genes were also modulated in mice upon microbial colonization. These genes were predominantly representing pathways involved proliferation, metabolism and (innate) immune responses, underpinning the conservation of the microbial colonization response across host species.

El Aidy et al. (2012, 2014) conventionalized adult germfree C57BL/6J mice and followed the host response as well as the microbial community over time. Interestingly, within one day after conventionalization, the bacterial log copy number reached it maximal size, not increasing further over the course of the experiment, indicating that a full-sized microbiota can establish quickly. In the first two days, the diversity remained low, but reached maximum levels around day 8 after conventionalization. Interestingly, at day 4 post conventionalization several known pathobionts (*Helicobacter, Sphingomonas*, and *Mucispirillum*) increase rapidly in abundance, coinciding with the highest activity of the innate immune system (measured by mucosal pro-inflammatory responses and plasma cytokine/amine levels). Subsequently, a sharp decrease in these pathobionts was observed between 8 and 16 days, coinciding with the increase of (innate and T cell-associated) immune activation and regulation (El Aidy et al., 2014). It can be hypothesized that microbial activation of innate immunity early in development creates a niche for species that can best resist this inflammatory environment (pathobionts), and subsequently their bloom induces adaptive immunity that in turn controls these species and restores the balance, thereby affecting microbial community structure.

Moreover, microbes can manipulate the host to gain a competitive advantage within the gut microbiome community. For example, *B. thetaiotaomicron* was shown to induce fucosylation of host surface glycans, which it can use as a substrate for growth (Bry et al., 1996; Hooper et al., 1999). Another example is given by Round et al. (2011), where it was shown that *Bacteroides fragilis* acts via the toll-like receptor (TLR)2 on T helper cells to ensure microbial symbiosis. Deletion of TLR2 specifically on T helper cells lead to activation of an anti-microbial response that limits *B. fragilis* colonization (for review of more host–microbe interactions by which microbes establish competitive advantages within the host’s gut microbiota, see Round et al., 2011; Stevens et al., 2021). Likewise, some pathogenic species have been shown to benefit from the host immune response eliminating their competitors. One illustrative example was given in a competition experiment using mice, *Haemophilus influenzae* colonization of the nasopharynx induced recruitment of host neutrophils and stimulated killing of complement-opsonized *Streptococcus pneumoniae*. This increase in neutrophils was not seen when mice were colonized by *Haemophilus* alone (Lysenko et al., 2005). This last study, like other similar studies showing interhost competition, also illustrates the caveat of studying cell–cell interactions looking at one or a few selected species, compared to investigating an entire microbial community of several hundred species (Scales et al., 2016; Wang et al., 2020; Kern et al., 2021). The net results of all these interacting bacteria, the environment and the host, might not be adequately modeled by studying interactions of each individual species in isolation.

Not only does microbial colonization of the intestines induce metabolic and immunological changes, it is well-established that this colonization is even necessary for the development of homeostatic metabolic and immune processes in the host. A prerequisite to be able to mount immune responses is the timing of colonization in relation to the development of the host. This is different when for example comparing mammals to fish. In mammals both innate and adaptive immune cells are present in the periphery at birth, while in fish only innate immune cells are present from 2 dpf (at time of hatching). The first adaptive cells (T cells) leave the thymus from 10 dpf onward. Adaptive immunity in fish is thought to be fully mature around 3–4 weeks post fertilization (wpf) when antibody responses can be measured, although more research is currently ongoing on the exact timing in different fish species (Lam et al., 2004; Brugman, 2016; Dee et al., 2016). Of interest is to understand the timing of immune maturation in relation to the colonizing microbes (e.g., different cells will be present at different life stages of the host). The zebrafish is especially suited to understand how innate and adaptive immune processes orchestrate the microbial composition in the intestine. Especially, the sequential development of innate and adaptive immunity develop enables investigation of the influence of early innate responses in the absence of peripheral (intestinal) adaptive immunity until 10–14 days post fertilization.

Taken together, the microbial composition in the gut is dynamic, especially during early life. The dynamic interactions between microbes and the host over time stimulates adaptation (gene regulation) and possibly evolution (loss or gain of functions) of the microbes (and eventually also the host, albeit at slower pace). During the lifetime of the host, regulatory processes ensure mucosal homeostasis. Regulatory cytokines IL-10 and IL-22, the focus of this review, are important factors in the maintenance of mucosal homeostasis between microbes and the host in the intestine. Aberrant signaling of these cytokines is often seen in (human) inflammatory disorders of the gut, such as inflammatory bowel disease (IBD) (Engelhardt and Grimbacher, 2014; Keir et al., 2020). For example, individuals with a missense mutation in either their IL-10 gene or its receptor genes my develop very early-onset IBD (Glocker et al., 2009; Kotlarz et al., 2012; Shouval et al., 2014). Next to IL-10, it is well known that polymorphisms in the IL-23R gene are associated with IBD (Neurath, 2019). As IL-23 signaling is also required for IL-22 induction, these polymorphisms might also implicate disturbances of IL-22 signaling in IBD susceptibility. Since IL-22 is both involved in regulatory as well as inflammatory processes, its precise role during IBD onset, flares and remission are currently unknown, but have been reported to be altered in IBD patients (Schmechel et al., 2008). In the next sections, the role of IL-10 and IL-22 in microbiota control is discussed.

## Function of IL-10 in Controlling the gut Microbial Community

IL-10 is produced by a variety of immune cells, of which major IL-10 producers include dendritic cells, macrophages, innate lymphoid cells (ILC2s) and regulatory T cells, and to a lesser extent by neutrophils, B cells, other T lymphocyte-subsets and even epithelial cells (Izcue et al., 2006; Saraiva and O’Garra, 2010; Ueda et al., 2010; Rivollier et al., 2012; Olszak et al., 2014; Seehus et al., 2017; Mishima et al., 2019; Miyamoto et al., 2019; Bando et al., 2020). IL-10 exerts its action by binding to the heterodimeric IL-10 receptor, consisting of IL-10R1, expressed on immune and other hematopoietic cells, and IL-10R2, which is widely expressed and can also bind to other cytokines such as IL-22 (co-receptor IL-22R1). Binding of IL-10 to its receptor activates Jak1 and Tyk2 and subsequently leads to phosphorylation of STAT3. In turn, STAT3 induces Socs3 expression, that inhibits pro-inflammatory cytokine pathways (Donnelly et al., 1999; Riley et al., 1999; Fillatreau and O’Garra, 2014). In this way IL-10 suppresses chemokine expression and downregulates co-stimulatory molecules [CD80, major histocompatibility complex (MHC) II] (Kasama et al., 1994; Willems et al., 1994; Wei et al., 2020). Furthermore, IL-10 decreases pro-inflammatory cytokine expression by inhibiting transcription factor NF-kB (Driessler et al., 2004). IL-10 also promotes the induction and maintenance of regulatory T cells (Tregs). Specifically, Tregs are induced when antigen presentation by dendritic cells occurs concomitantly with an instructive IL-10/TGF-β milieu (Izcue and Powrie, 2008). In addition, IL-10 maintains Foxp3 expression (a master regulator in the development and function of Tregs) in these cells during inflammation. These Tregs may further suppress inflammatory responses (Izcue et al., 2006; Murai et al., 2009). IL-10–/– mice develop spontaneous colitis (Kühn et al., 1993). However, neither germfree IL-10–/– mice nor their derivatives that are mono-associated with certain pathobionts (e.g., *H. hepaticus*) develop this disease, suggesting that colonization with a complex microbiota may be a prerequisite for disease development in these mice (Sellon et al., 1998; Dieleman et al., 2000). Intriguingly, various studies suggested that differences in the composition of such complex microbiota strongly affects disease severity (Kim et al., 2005; Sydora et al., 2005; Büchler et al., 2012; Yang et al., 2013).

Several studies reported differences in gut microbiota composition between mice that lack IL-10 and their wildtype counterparts (summarized in Table 1). Multiple factors may confound data on microbiota composition, indicating the importance of controlling for these factors as much as possible. Firstly, given the importance of environmental stimuli in shaping the microbiota, it is important to co-house experimental animals to eliminate cage- or tank-specific effects. Secondly, to minimize the effect of different genotypes (ensuring the genetic variation is only in the gene of interest) the use of littermates would be ideal (for a comprehensive review, see Laukens et al., 2016). Differences in gut microbiota composition were demonstrated by conventionalization of adult germfree IL-10–/– and WT littermates that led to distinct microbiota development in these animals (Maharshak et al., 2013). While the microbiota diversity and richness decreased over time in the IL-10–/– mice, this was not seen in WT littermates. In contrast, WT littermates displayed an increasing diversity until the final timepoint analyzed at 2 weeks post conventionalization (Maharshak et al., 2013). Specifically, the abundance of Proteobacteria (*E. coli*) increased over time in the conventionalized IL-10–/– mice, which coincided with the activation of spontaneous inflammation and the onset of colitis. Analogously, increased *E. coli* abundance was also observed in IL-10–/– mice that were conventionally raised at a timepoint that colonic inflammation was also apparent (at 8–10 weeks of age) in these animals (Maharshak et al., 2013). Unfortunately, the microbiota composition of these conventionally raised animals was not investigated at earlier ages when inflammation was still absent, disallowing the analysis of the time-dependent co-development of the microbiota and the inflammatory responses during early life stages. Such studies could reveal microbiota changes that occur prior to the onset of spontaneous colitis, while the present study observed differences in microbial composition between wildtype and IL-10–/– littermates may very well be a consequence of the inflammatory milieu that selects for bacteria that can resist such conditions, like *E. coli* (Thiennimitr et al., 2011). Moreover, in the latter experiments the IL-10–/– mice and WT littermates were not co-housed, and the differences detected may partially depend on cage effects. Several studies confirm the elevated *E. coli* abundance in IL-10–/– mice (Arthur et al., 2012, 2014; Overstreet et al., 2021). Unfortunately, these studies also do not include samples taken prior to inflammation onset, and do not compare between co-housed IL-10–/– and wildtype littermates, which disables the exclusion of several environmental factors that can confound the microbiota comparisons. However, prolongation of the analyses of the IL-10–/– mice associated microbiota to later ages established that in both the wildtype and IL-10–/– mice the abundance of *E. coli* went down over time, albeit that at each timepoint the *E. coli* abundance was higher in the IL-10–/– mice compared to their wildtype counterpart (Arthur et al., 2012, 2014). Taken together these findings suggest that the initial colonization in WT mice with higher levels of *E. coli*, followed by microbiota succession toward a more Firmicutes and Bacteroides dominated microbiota, is also occurring in IL-10–/– animals although at a slower rate. None of these studies is able to decide whether the observed higher abundance of *E. coli* plays a causative role in the increasing inflammation in the IL-10–/– mice or is the consequence of the inflammatory milieu. The delayed microbiota maturation toward a Bacteroidetes and Firmicutes dominated ecosystem in the IL-10–/– mice, could suggest that the IL-10–/– mice might have difficulty regulating/controlling Enterobacterial species. Their initial colonization is likely to trigger a state of ‘transient’ inflammation in the wildtype mice that is balanced by regulatory responses that establish appropriate homeostatic relations with typical intestinal microbial phyla of the Bacteroidetes and Firmicutes, while suppressing enterobacterial species, such as *E. coli* (Renz et al., 2011; Al Nabhani et al., 2019). This secondary regulatory response is absent in IL-10–/– mice, which may lead to intestinal inflammation directly and may eventually drive toward colitis. Intriguingly, a recent report showed that IL-10–/– mice contain more immature granules in their Paneth cells, suggesting a role for IL-10 in Paneth cell function modulation (Berkowitz et al., 2019). Especially since Paneth cells are the predominant producers of anti-microbial peptides that play an important role in keeping the bacteria away from the replicating stem cell niche. Disturbed secretion of antimicrobial factors might explain (part of) the observed loss of microbial containment in the IL-10–/– intestine. In addition, it was also shown that although the inner mucus layer in the colon seemed to be even thicker in IL-10–/– mice, it was shown to be more penetrable by bacteria compared to that of wildtype mice (Johansson et al., 2014).

**TABLE 1:** Summary of studies investigating the role of lack of IL-10 on the development of colitis and microbiota composition.

	Animal model	Intervention	Analysis	Timeline	Littermates/Co-housed	Findings	References
**IL-10^–/–^**	Mouse	Conventionally and SPF raised mice	Development of colitis	Analysis at 0–12 weeks of age	Littermates, not co-housed	IL-10^–/–^ mice develop spontaneous colitis	Kühn et al., 1993
	Mouse	GF, SPF and colitis-related-bacteria-colonized mice	Development of colitis	GF: 0–6 months SPF: 0–38 weeks Colitis-related-bacteria-colonized: adults; analysis 5–32 weeks post-colonization	Littermate status not clear, not co-housed	Development of spontaneous colitis in IL-10^–/–^ mice is dependent on the presence of microbiota	Sellon et al., 1998
	Mouse	Mono-colonization with *Helicobacter hepaticus*	Development of colitis	2-month old mice; analysis 7–16 weeks post-colonization	Littermates; not co-housed	*H. hepaticus* mono-colonization did not induce colitis in IL-10^–/–^ mice	Dieleman et al., 2000
	Mouse	SPF; in different facilities; infection with *H. hepaticus*	Microbiota composition and susceptibility to colitis severity	Analysis at 38, 141, 172, and 204 days of age	Not littermates; not co-housed	Susceptibility to *H. hepaticus*-induced colitis differed among facilities, related to differing microbiota compositions	Yang et al., 2013
	Mouse	Mono-colonization with *H. hepaticus* and SPF infected with *H. hepaticus*	Microbiota composition and susceptibility to colitis severity	8-week old mice; analysis 4 and 13 weeks post-colonization	Not co-housed	Changes in gut microbiota composition are involved in strain-specific susceptibility of IL-10^–/–^ mice to colitis	Büchler et al., 2012
	Mouse	Mono-colonization with specific bacterial species	Susceptibility to colitis severity	Analysis 7 and 22 weeks post-colonization	Not littermates; not co-housed	Bacterial mono-colonization did not induce colitis in IL-10^–/–^ mice, despite stimulating a systemic response	Sydora et al., 2005
	Mouse	Mono-colonization with commensal bacterial species	Susceptibility to colitis severity	10–12 weeks old mice; analysis 1–46 weeks post-colonization	Not littermates; not co-housed	Different commensal bacteria initiate different patterns of intestinal inflammation	Kim et al., 2005
	Mouse	Embryonic transfer into dams differing in microbiota composition; infection with *H. hepaticus*	Microbiota composition and disease severity	Infection at 24–26 days of age; analysis at 111 days of age;	Littermates, co-housed and maternal microbes tested	Disease severity can be influenced by microbiota composition, which depends on maternally inherited gut microbiota and host genotype	Hart et al., 2017
	Mouse	(1) GF IL-10^–/–^ and WT colonized with same donor microbiota; (2) SPF raised IL-10^–/–^ and WT	Microbiota composition	(1) Adults; analysis 1–4 weeks post-colonization (2) Analysis at 8 and 10 weeks	Littermates; not co-housed	Over time, WT mice had increased gut microbiota diversity and richness, IL-10^–/–^ mice had decreased diversity and richness, with increase in Proteobacteria (*Escherichia coli*). SPF raised mice showed similar increase in *E. coli*	Maharshak et al., 2013
	Mouse	SPF IL-10^–/–^ and WT	Microbiota composition	Analysis at 4–19 weeks of age	Not littermates; not co-housed	Microbiota diversity was similar between young WT and IL-10^–/–^ mice, but IL-10^–/–^ mice did not show increased microbial complexity as WT did, together with an increase in *E. coli* and a reduction in *Clostridium* in IL-10^–/–^ mice	Overstreet et al., 2021
	Mouse	GF IL-10^–/–^ and WT colonized with SPF microbiota	Microbiota composition	Analysis at 2–20 weeks post-colonization	Not littermates; not co-housed	Increase in *E. coli* in IL-10^–/–^ mice at 2 weeks; *E. coli* abundance decreased over time in IL-10^–/–^ and WT, but remaining higher in IL-10^–/–^	Arthur et al., 2012, Arthur et al., 2014
	Mouse	(1) SPF IL-10^–/–^ and WT (2) SPF IL-10^–/–^ and WT treated with antibiotics	Paneth cell function	(1) analysis at 7 weeks old (2) 9 weeks old mice; analysis at 11 weeks	Not littermates; not co-housed	More immature granules in Paneth cells of IL-10^–/–^ mice. Without microbiota, Paneth cells were more mature, but with more amorphous granules	Berkowitz et al., 2019
	Mouse	IL-10^–/–^ and WT	Bacterial penetration/localization	Analysis at 8–12 weeks of age	Not littermates, not co-housed	More penetrable mucus layer and direct bacteria-epithelium contact in IL-10^–/–^ mice	Johansson et al., 2014
	Zebra-fish	IL-10^–/–^ and WT	Development of colitis	Analysis at 1 year old	Siblings; not co-housed	IL-10^–/–^ zebrafish do not show any signs of intestinal inflammation at baseline	Harjula et al., 2018
	Mouse	IL-10–/–IL-22–/– and IL-10–/–	Development of colitis and microbiota composition	16–20 weeks old mice	Not littermates; not co-housed	IL-22 deficiency prevents spontaneous colitis in IL-10–/– mice Both IL10–/– and IL-10–/–IL-22–/– display more Th17 cells. Microbial diversity lower in IL-10–/– but same as wildtype in IL-10–/–IL-22–/– mice	Gunasekera et al., 2020
IL10R–/–	Mouse	SPF IL-10Rb(IL-10R2)^–/–^ and IL-10Rb(IL-10R2)^±^	Microbiota composition	Analysis at 0–30 weeks of age	Littermates; co-housed	Inflammation is dependent on microbiota in IL-10Rb^–/–^ mice; No difference in microbiota composition and diversity between IL-10Rb^–/–^ and IL-10Rb^±^	Redhu et al., 2017

*GF, germfree; IL, interleukin; SPF, specific pathogen free; WT, wildtype.*

Interestingly, Fung et al. (2016) showed that a population of ‘lymphoid resident bacteria’ (specific members of the α-proteobacteria and β-proteobacteria) exist that are able to colonize and survive in murine dendritic cells. Dendritic cells colonized with members of these lymphoid resident bacteria expressed higher levels of IL-10 (and IL-1β and IL-23) compared to uncolonized dendritic cells. In the absence of IL-10, an almost 10-fold increase in Th17 + cells in the mesenteric lymph nodes and Peyer’s patches was observed, which might indicate that IL-10 induced in the lymphoid tissues by these lymphoid tissue resident commensals (inside dendritic cells) is able to directly limit Th17 expansion and create a tolerogenic environment in the lymphoid tissues (Fung et al., 2016).

Arguing against a role for IL-10 signaling in shaping the microbial composition is the observation that IL-10 receptor b–/– (IL-10rb–/–) mice did not differ in their microbial composition and diversity from their littermates (IL-10rb+/–) (Redhu et al., 2017). Yet, the authors do describe that a few significant minor operational taxonomic units (OTU) differences were observed at different ages of the animals, however, these were not further specified. An important difference with the other studies described above is that the mice used in this study were littermates that were co-housed in a specific pathogen free facility.

In an effort to unravel the link between host genotype, environmental factors and severity of colitis in IL-10–/– mice, Hart et al. (2017) performed an embryonic transfer study. IL-10–/– embryos were implanted in surrogate dams which were allowed to naturally deliver and raise the pups. IL-10–/– pups born from surrogate mothers with different backgrounds developed a different microbiota and displayed different disease susceptibility when subsequently colonized with *H. hepaticus.* In this study IL-10–/– embryos were taken from different background [C57BL/6 (B6) and C3H/HeJBir (C3H)] and surrogate dams could be harboring the Charles River, Taconic farms or Jackson farms microbiota. As has been shown previously, animals derived from these different farms harbor a different microbiota. This has consequences for the presence (percentage) of different immune cell subsets in the intestines (Ivanov et al., 2009). Interestingly, in the B6 IL-10–/– mice increased microbial diversity was found in those pups born from mothers harboring the Charles River microbiota and these pups also had the lowest disease score after colonization with *H. hepaticus* compared to the B6 IL-10–/– mice raised by mothers harboring Taconic or Jackson farm microbiota. On the C3H background, IL-10–/– mice born to surrogate dams from Jackson or Taconic farms had increased disease severity (but also increased microbial diversity) compared to those born from surrogate dams harboring Charles River microbes. For both C3H and B6 IL-10–/– mice, those raised by Charles River containing surrogate dams had the lowest relative abundance of *H. hepaticus*. So this study illustrates that disease severity and microbial composition (including the ability of the disease causing *H. hepaticus* to colonize) depends on the gut microbiota of the surrogate dams as well as on the genetic background of the pups (Hart et al., 2017). This last study really emphasizes the need to use littermates and to co-house the animals studied, especially when looking at dynamic and interactive processes such as colonization and immune development.

In conclusion, the impact of IL-10 on the composition of the microbiota is far from clear and properly controlled experiments are needed. Sampling should be performed over time, including early time points that precede the onset of colitis, and employing littermates (IL-10+/+ and IL-10–/–) that are co-housed. One could expect an effect of IL-10 on microbial composition, if only based on the fact that IL-10 plays a role in the maintenance and development of IgA + plasma cells that are reported to have an impact on the microbial composition (Kunisawa et al., 2013; Huus et al., 2021). This might indicate that timing of sampling is crucial, since developing intestinal inflammation in these IL-10–/– mice might obscure more subtle dysbiotic processes early on. To investigate early innate regulation in the absence of adaptive immunity, the zebrafish model might be useful, as zebrafish develop from eggs *ex utero* and solely rely on innate immunity in the first 1–2 weeks of life (Brugman, 2016; Dee et al., 2016). Notably, mutant zebrafish lacking functional IL-10 did not develop spontaneous colitis, but did express increased levels of interferon (IFN)-γ in the gills (Harjula et al., 2018; Bottiglione et al., 2020). Moreover, inflammatory responses in the gills upon exposure to irritants (Resiquimod R848) were stronger in IL-10 deficient zebrafish compared to wildtypes, although this difference could only be shown in later phases of the inflammatory response (Bottiglione et al., 2020). Since colitis in IL-10–/– mice is clearly associated with presence of certain members of the microbiota, further investigation into the microbial composition and possible disease inducing potential of (specific or combinations of) microbes in IL-10–/– zebrafish could shed some light on loss or gain of IL-10 and the effect on the microbial composition in health and disease.

Next to microbial composition analysis, determinants of host barrier function (Paneth cell function, epithelial proliferation and mucus structure) should be investigated in parallel, especially at early time points that precede full-blown inflammation, in order unravel the multiple factors contributing to dysbiosis and loss of homeostasis in IL-10–/– animals. An important factor in epithelial barrier function regulation is IL-22. In the next section the role of IL22 in keeping the peace at the mucosal surface is addressed.

## Function of Interleukin 22 in gut Microbial Composition

IL-22 belongs to the IL-10 family of cytokines, together with IL-19, IL-20, IL-24 and in humans, IL-26 (Parks et al., 2015; Shohan et al., 2020). Production of IL-22 occurs particularly at mucosal surfaces. While many cells like macrophages, neutrophils, CD4^+^ Th17, Th22, γδ T cells, LTi cells, and natural killer T cells have all been shown to produce IL-22, group 3 ILCs are probably its major innate cellular source (Liang et al., 2006; Cella et al., 2009; Colonna, 2009; Eyerich et al., 2009; Ouyang et al., 2011; Rutz and Ouyang, 2011; Hansson et al., 2013; Zindl et al., 2013; Yeste et al., 2014; Dudakov et al., 2015).

IL-22 exerts its actions via binding to the IL-22 receptor, which consists of two subunits: IL-22R1 and IL-10R2 (Dumoutier et al., 2000; Xie et al., 2000; Kotenko et al., 2001a,b; Li et al., 2004). While IL-22 is mainly produced by immune cells, it is targeting non-immune cells; in the intestines IL-22R is expressed mainly on epithelial cells (Shohan et al., 2020). In general, IL-22 promotes antimicrobial activity by stimulating antimicrobial peptide production by epithelial cells, inducing mucus production and supporting tissue repair (Radaeva et al., 2004; Sugimoto et al., 2008; Zheng et al., 2008). Furthermore, IL-22 may also promote intestinal stem cell (ISC) expansion (Lindemans et al., 2015).

IL-22 has been reported to have both regulatory and inflammatory effects depending on the context. Low level IL-22 production and signaling (during homeostasis) seems to strengthen epithelial barrier function, while increased levels of IL-22 induced during inflammation help clearing bacterial infections, such as shown for *Citrobacter rodentium* infection (Munoz et al., 2015; Tsai et al., 2017). Secreted IL-22 receptor, IL-22 binding protein (IL-22BP) (produced by dendritic cells) is upregulated during inflammation and might be a regulator of overt inflammatory effects (reviewed in Parks et al., 2015). In the next section, we will focus on the microbiota shaping potential of IL-22. An overview of studies making use of IL-22–/– animals is given in Table 2.

**TABLE 2:** Summary of studies investigating the role of lack of IL-22 on the microbiota composition.

	Animal model	Intervention	Analysis	Timeline	Littermates/Co-housed	Findings	References
**IL-22^–/–^**	Mouse	SPF IL-22^–/–^ and WT	Microbiota composition and susceptibility to colitis severity	Start DSS treatment: 8–12 weeks of age; start co-housing: 4–6 weeks pre-DSS treatment; analysis 1 week post-DSS initiation	Not littermates and littermates; separately and co-housed	IL-22–/– more susceptible to colitis induction. Transfer of IL-22–/– microbes to WT in co-housing. IL-22 seems to shape balance between immunity and colonic microbiota	Zenewicz et al., 2013
	Mouse	Ltbr^–/–^ (lack IL-23 and IL-22) and WT	Microbiota composition	Analysis at 13 weeks of age	Littermates	Lack of IL-22 and IL-23 was associated with an increase in small intestinal epithelial-attaching SFB	Upadhyay et al., 2012
	Mouse	SPF Ahr^–/–^ (less IL-22 production)	Microbiota composition	Analysis at 6–10 weeks of age	Littermates	Ahr^–/–^ mice show increased epithelial-attaching SFB	Qiu et al., 2013
	Mouse	IL-22^–/–^, Rag^–/–^/IL-22^–/–^ and WT; inoculation with *Achromobacter* + antibiotics treatment	Bacterial lymphoid tissue colonization	6–12 weeks of age; analysis 10 days post inoculation	Co-housed or littermates	Lack of IL-22 prevented *Achromobacter* colonization of lymphoid tissue	Fung et al., 2016
	Zebra-fish	Morpholino knock-down of IL-22; exposure to *Aeromonas hydrophila* or LPS	Susceptibility to bacterial infection	*A. hydrophila* infection at 2 dpf; analysis at 3 dpf	Siblings, not co-housed	Knock-down of IL-22 increased mortality in absence of infection; exposure to *A. hydrophila* or LPS increased pro-inflammatory cytokine expression and reduced survival with IL-22 knock-down	Costa et al., 2013
	Mouse	SPF Rag^–/–^ (no B and T cells) mice with ILC depletion/anti-IL-22 mAb treatment; administration of IL-22	Microbial translocation	Adult	n.a.	Lack of ILCs led to bacterial translocation of lymphoid tissue resident *Alcaligenes* and systemic inflammation, which could be prevented by IL-22 administration	Sonnenberg et al., 2012
	Mouse	SPF atherosclerosis-prone IL-23^–/–^, IL-22^–/–^ (via bone marrow reconstitution); fed WD	Microbiota composition	Start experiments: 6 week old; analysis 8–16 weeks post-WD feeding	Littermates; co-housed	Inactivation of IL-23-IL-22 signaling reduced intestinal barrier function and increased epithelial adherent bacteria and increased systemic LPS	Fatkhullina et al., 2018
	Mouse	IL22-antibody treatment	Microbiota composition during *C. difficile* infection	Rag1–/–, WT, 8- to 16-week-old female and male mice	No littermates, not co-housed	IL-22 modulated glycosylation promoted the growth of succinate consuming bacteria which sequestered the succinate from use by *C. difficile*, preventing outgrowth of *C. difficile* and disease in the intestine	Nagao-Kitamoto et al., 2020
	Mouse	IL-10–/–IL-22–/– and IL-10–/–	Development of colitis and microbiota composition	16–20 weeks old mice	Not littermates; not co-housed	IL-22 deficiency prevents spontaneous colitis in IL-10–/– mice Both IL10–/– and IL-10–/–IL-22–/– display more Th17 cells. Microbial diversity lower in IL-10–/– but same as wildtype in IL-10–/–IL-22–/– mice	Gunasekera et al., 2020
**IL22R–/–**	Mouse	IL22R1–/–	Antimicrobial effector functions during *C. rodentium* infection/DSS and microbial composition	Adult mice DSS 7 days *C. rodentium* 10–25 days post infection	No littermates, not co-housed	IL-22R signaling protected against lethal *Citrobacter rodentium* infection by increasing colonization resistance to other disease-enhancing pathobionts such as *E. faecalis*	Pham et al., 2014
	Mouse	SPF paneth cell specific IL-22R1^–/–^	Antimicrobial effector functions and microbial composition	Analysis at 6–8 weeks of age	Littermates; co-housed	Paneth cell antimicrobial effector function depends on IL-22Ra1 signaling. Paneth cell specific IL-22R deletion was associated with an altered microbial composition (increased SFB)	Gaudino et al., 2021

*Ahr, aryl hydrocarbon receptor; dpf, days post fertilization; DSS, dextran sodium sulfate; IL, interleukin; ILC, innate lymhoid cells; LPS, lipopolysaccharide; Ltbr, lymphotoxin beta receptor; mAb, monocloncal antibody; SFB, segmented filamentous bacteria; SPF, specific pathogen free; WD, western diet; WT, wildtype.*

An important role for keeping the peace via IL-22 might be via innate lymphoid cell (ILC) function. For example, in the absence of ILCs, *Alcaligenes* species could disseminate (and cause systemic disease) in mice, which could be prevented by the administration of IL-22 (Sonnenberg et al., 2012). Furthermore, IL-22 has been shown to induce the proliferation of epithelial cells, strengthening the intestinal barrier (Ouyang et al., 2011; Lindemans et al., 2015). Recently, it was shown that by using Paneth cell-specific IL-22Rα (IL22R1)–/– mice, that Paneth cell maturation and antimicrobial effector function are dependent on IL-22R signaling (Gaudino et al., 2021). C57BL/6 Paneth cell specific IL-22R–/– (IL-22Ra1^fl/fl^;Defa6-cre) mice displayed reduced expression of Paneth cell-specific Lyz1, Mmp7, and some α-defensins.

Since IL-22 induces antimicrobial peptides and mucins it is likely that IL-22 has intestinal health and microbiota modulating effects (Wolk et al., 2004; Liang et al., 2006; Wolk et al., 2006; Boniface et al., 2007; Sugimoto et al., 2008; Zheng et al., 2008). Indeed, it was shown that α-IL-22 antibody administration to germfree mice colonized with a human microbiota changed the composition of the gut microbiota (Nagao-Kitamoto et al., 2020). Further research showed that IL-22 modulated glycosylation of host N-linked glycans in this model. In this humanized microbiota mouse model, these host glycans promoted the growth of succinate consuming bacteria which sequestered the succinate from use by *C. difficile*, preventing outgrowth of *C. difficile* and disease in the intestine (Nagao-Kitamoto et al., 2020). This microbiota modulating effect by IL-22 via effects on glycosylation was also shown in IL22R–/– mice. Although no differences in microbiota composition were observed between IL22R–/– mice and wildtype mice at baseline, it was shown that IL-22R signaling protected against lethal *Citrobacter rodentium infection* by increasing colonization resistance to other disease-enhancing pathobionts such as *E. faecalis* (Pham et al., 2014). It was shown that IL22R signaling promotes the expression of Fut2, required for epithelial cell surface fucosylation. This fucosylation has been shown to shape the intestinal microbiota by favoring colonization of some (beneficial) species, while preventing others (pathobionts) like *E. faecalis* to colonize (colonization resistance) (Marcobal et al., 2011). Administration of fucosylated oligosaccharides to *C. rodentium-*challenged IL-22R–/– mice decreased the colonization of *E. faecalis* and attenuated *C. rodentium* infection (Pham et al., 2014). Also, administration of fucosylated oligosaccarides increased the diversity and composition of the microbiota in IL-22R–/– mice during *C. rodentium* infection. Therefore, it seems that IL22 signaling is able to restore the diversity of anaerobic commensal symbionts by increasing *Fut2*-mediated fucosylation on epithelial cells thereby favoring colonization of beneficial bacteria.

The aforementioned Paneth cell specific IL-22R–/– mice, that show decreased anti-microbial peptide production, also showed an altered microbial composition (Gaudino et al., 2021). 16S DNA sequencing of co-housed littermates revealed increased colonization of α-Proteobacteria and Peptostreptococcaceae in the terminal ileum of Paneth cell specific IL-22R–/– mice. Quantitative PCR showed an increase in epithelial cell attaching segmented filamentous bacteria in the ileum of these mice. The fact that in the absence of IL-22, the host is unable to prevent epithelial attachment has been shown consistently. For example, mice lacking the lymphotoxin β receptor (which have decreased IL23 and IL-22 production) and Ahr–/– mice (in which ILC3s produce less IL-22), all displayed an increased levels of epithelial-attaching segmented filamentous bacteria (Upadhyay et al., 2012; Qiu et al., 2013). Likewise, low density lipoprotein receptor (ldlr) knock out mice (model for atherosclerosis) with a deletion of either IL23 or IL-22 in the bone-marrow derived cells (immune cells) exhibited reduced intestinal barrier function and increased epithelial adherent bacteria including Clostridiaceae and Ruminococcaceae, causing systemic increase in lipopolysaccharide (LPS) and trimethylamine *N*-oxide (TMAO) associated with development of atherosclerosis (Fatkhullina et al., 2018). On the other hand, pathogens are sometimes able to use the induction of antimicrobial protein expression induced by IL-22 to their advantage. It has been shown that antimicrobial proteins, such as lipocalin-2 and calprotectin, can sequester essential metal ions from microbes, thereby inhibiting their growth (Behnsen et al., 2014). However, some pathogens that can overcome this metal ion starvation can benefit and make use of this competitive advantage (Behnsen et al., 2014). IL-22-induction of antimicrobial peptides therefore does not automatically lead to homeostasis, but is influenced by the continuous evolutionary rat race between host and pathogens.

While several studies show different mechanisms by with IL-22 might modulate certain species within the microbiota, Zenewicz et al. (2013) investigated whether IL22–/– mice displayed differences in the total gut microbiota by also controlling for host genetic background and environmental effects, something that most studies neglect. While IL-22–/– mice did not show different colonic architecture compared to wildtype mice, they do develop more severe colitis in response to dextran-sodium sulfate (DSS) treatment compared to wildtype mice (Zenewicz et al., 2013). Interestingly, cohousing wildtype mice with IL-22–deficient mice increased the severity of DSS-induced colitis in these wildtype mice, suggesting that the colitis might be ‘transmissible’ as has been shown for TRUC mice (mice that are deficient for both T-bet and Rag2) (Garrett et al., 2007). This suggests that transmissible agents, most likely (members of) the microbiota can ‘transmit’ this increased severity. The IL-22–/– mice still developed more severe colitis upon DSS treatment than the wildtype mice when co-housed, and therefore both the lack of IL-22 function in the host, as well as the (‘transmissible’) microbiota of these mice play a role in the severity of DSS-induced colitis. To tease apart the effects of host genotype and housing, heterozygous siblings (IL-22+/–) were in crossed to obtain a colony of wildtype, heterozygous and mutant IL-22 mice, in which the mice were siblings and co-housed and colitis was induced by DSS (Zenewicz et al., 2013). In this sibling and co-housing controlled experiment, again the wildtype mice co-housed with the IL-22–/– showed increased wasting disease compared to wildtype not co-housed with their IL-22–/– siblings (Zenewicz et al., 2013). Fecal samples were taken of these mice prior to DSS treatment and the microbial composition was determined by 16S sequence analysis. Interestingly, while the wildtype siblings housed alone harbored a significantly different microbiota compared to their IL22–/– siblings, this difference between IL22–/– and wildtype was completely lost upon co-housing. Here, the co-housed wildtype and IL22–/– mice both had lower abundance of *Lactobacillus*, *Bacteroides*, *Ruminococcus*, *Turicibacter*, *Anaerobacter, Parabacteroides*, and *Hespellia* and higher abundance of *Coprococcus, Allobaculum, Barnesiella, Alistipes, Xylanibacter, Butyricimonas*, and *Helicobacter* compared to the wildtype siblings housed alone (Zenewicz et al., 2013).

Intriguingly, the wildtype mice that were co-housed for four weeks with the IL-22–/– siblings showed a reduced expression of RegIIIγ and RegIIIβ in the colon compared to their wildtype siblings that were separately housed (Zenewicz et al., 2013). So, it seems that the microbes of IL-22–/– mice were transmitted to wildtype litter- and cage-mates, and in turn were able to influence the levels of antimicrobial peptides and DSS colitis severity in these wildtype mice. How these microbes upon transmission find a niche and are able to establish themselves in immune-competent wildtype mice, let alone subsequently have an effect on anti-microbial peptide production in these wildtype mice is as yet unclear, but warrants further investigation. Using mice conventionalized at neonatal or adult age with these ‘colitis transmitting’ microbes (from mice or from human IBD patients) might lead to an understanding of the underlying mechanisms.

Interestingly, it seems that IL-22 is not solely keeping bacteria clear from the epithelial surface and favoring species to colonize the gut lumen, but might also ensure lymphoid tissue colonization (Fung et al., 2016). It was shown that IL-22 derived from ILC3 cells played a role in the colonization of lymphoid tissues by certain species. By inoculating IL-22–/–, Rag–/–IL-22–/–, WT and IL17a–/– mice with one of the species that have been shown to colonize lymphoid tissues, *Achromobacter*, it was shown that the mice lacking IL-22, lacked *Acromobacter* colonization of lymphoid tissue (Fung et al., 2016). It seems, therefore, that IL-22 modulates the microbiota giving some species (able to colonize lymphoid tissue) a competitive advantage. How this permissive effect of IL-22 on microbes colonizing the lymphoid tissues in mice can be explained in the context of its clear role in maintaining epithelial barrier integrity, protecting against unwanted epithelial-attachment and translocation by increasing mucus and antimicrobial peptide production is currently unclear. This however, does link back to the previous study by Zenewicz et al. (2013), in which it was shown that IL-22–/– microbes were able to colonize wildtype cage-mates and where able to influence antimicrobial peptide production (reduced RegIIIβ and RegIIIγ) in these wildtype hosts.

In the study of Fung et al. (2016) the observed colonization of lymphoid tissue by certain bacterial species (members of the α-proteobacteria and β-proteobacteria) appeared to be important for the protection against DSS colitis by their induction of IL-10 (and IL-22). It might be interesting to study these processes early in life of the mice. Does lymphoid tissue colonization occur naturally and is IL-22 involved in this process during early natural colonization? Does this occur in other species than laboratory mice as well? For example, it might also be interesting to investigate whether this process of IL-10 induction through colonization of dendritic cells also exists in those species that lack mucosal-associated lymphoid tissues in the intestines, like fish, or whether this is an evolutionary adaptation that evolved with the development of lymph nodes draining the intestine.

IL-22 is conserved in zebrafish (Igawa et al., 2006; Siupka et al., 2014) and also found in other fish species (Corripio-Miyar et al., 2009; Monte et al., 2011; Costa et al., 2013; Yang et al., 2020). In zebrafish, expression of IL-22 was seen in the intestine and gill at baseline and increased after a challenge with TLR agonists (LPS/Poly I:C) (Siupka et al., 2014). When IL-22 was injected to mpx:GFP/lysC:DsRED2 double transgenic fish (staining myeloperoxidase-producing cells green and granulocytes red, respectively) it induced GFP expression specifically in enterocytes (Siupka et al., 2014). It has been shown that next to neutrophils, enterocytes also express myeloperoxidase during infection (Rendueles et al., 2012). Thus, zebrafish IL-22 also seems to signal to gut epithelial cells, just like mammalian IL-22. Constructing IL-22–/–, IL-22R–/–, and IL-22BP–/– zebrafish might shed light on the effect of IL-22 on epithelial barrier function in these fish.

It has been shown using zebrafish that knock-down of IL-22 by morpholino in larval zebrafish in the absence of bacterial infection, tended to increase mortality (Costa et al., 2013). During bacterial bath exposure to *Aeromonas hydrophila* or LPS, the IL-22 knock down larvae showed increased severity of disease; they displayed higher levels of *tnfa* and *il1b* expression and reduced survival (Costa et al., 2013). This might indicate that like in mammals, low levels of IL-22 are necessary to maintain homeostasis and increased levels might be necessary to combat infections.

Recently, ILC-like cells expressing *il22* have also been discovered in zebrafish using single-cell transcriptional analysis in rag1*-*deficient zebrafish (lacking B and T lymphocytes) (Hernandez et al., 2018). However, these cells differ from the mammalian counterparts. Specifically, zebrafish ILC-like cells were not found to constitutively express cytokine receptors which human and mouse ILCs do express, nor AhR and pattern recognition receptors (Hernandez et al., 2018). Instead, zebrafish ILC-like cells expressed novel immune-type receptors (NITRs), putative orthologs of mammalian natural cytotoxicity receptors (NCRs) and killer cell immunoglobin like receptors (KIRs) (Hernandez et al., 2018). Thus, these ILC-like cells in zebrafish may possibly resemble human and mice NCR + ILC3s in key ways, including IL-22 expression in response to bacterial infection. Nevertheless, these aforementioned receptor differences might have consequences for their regulation.

Besides ILC-like cells, other potentially conserved relevant factors regarding IL-10 and IL-22 signaling have also been discovered in the zebrafish genome, strengthening the comparisons that can be made between these fish and mammals. One such factor involved in IL-22 signaling is IL-23, that acts via STAT3 to stimulate IL-22 production in ILCs and T cells (Zheng et al., 2008; Guo et al., 2014). Both *il23a* (p19) and *il23r* have been annotated in the zebrafish genome as well as *il1b*. IL-23a and il1b expression has been shown to be upregulated in zebrafish colitis (Brugman et al., 2009; Oehlers et al., 2012). Whether *il23* and *il1b* induce *il22* production in zebrafish needs to be determined.

A word of caution in light of similarity of genes or cells of different species is worth giving here. As for similarity of genes between (host) species, it has to be noted that (automatic) annotation might not mean functional conservation as has been shown for the IL-10R2 (Piazzon et al., 2016). In terms of receptor similarity it is shown that for IL-10R1 functional homology exists between fish and mammals. Annotation of the IL-10R2 (the receptor that, when conserved, also might bind IL-22) has been confusing. Since the two putative IL-10R2 receptors (CRFB4 and CRFB5) are very similar in protein structure and genomic organization both are annotated in the zebrafish database^1^ as IL-10R2 (ENSDARG00000078042 and ENSDARG00000068711). Studies performed in grass carp actually showed that CRFB4 is probably the functional homolog of IL-10R (Wei et al., 2014; Piazzon et al., 2016). Additionally, the lack of cell markers for specific cell types in zebrafish sometimes leads to assumptions on the presence of certain cell types only on the basis of gene expression data. This should always be interpreted with caution.

The presence of IL-22BP in zebrafish has been predicted via phylogenetic analysis (Stein et al., 2007) and its cDNA has been cloned (Levraud et al., 2007). Recently, IL-22BP has been functionally characterized in mandarin fish *Siniperca chuatsi* (Huo et al., 2019). In this study, IL-22BP bound to IL-22 and thereby prevented the induction of target genes of IL-22. This suggests that at least in mandarin fish, IL-22 and IL-22BP are functionally conserved. However, whether IL-22 has the same microbiota modulating effects in (zebra)fish as in mammals during homeostasis needs to be further investigated.

In conclusion, studies performed in IL-22–/– mice are clearly showing microbiota modulating potential of IL-22. IL-22 induces antimicrobial activities, mucin production and increasing epithelial integrity, thereby preventing microbes to adhere and invade epithelial cell surfaces. However, at the same time IL-22 seems to allow certain bacteria to colonize lymphoid tissues necessary for IL-10 regulatory processes, by currently unknown mechanisms. Additionally, it is unclear why IL-10R2–/– mice do not show differences in their microbial composition, while this receptor subunit is also part of the IL-22R (IL-22R1, IL-10R2) and knock out of this subunit should also impair IL-22 signaling, shown to play a role in shaping the microbial composition. For example, Paneth cell specific IL-22R(1)–/– mice also showed an altered microbial composition (Gaudino et al., 2021). The fact that the IL-22Rα (IL-22R1) subunit also binds IL-20, IL-22, and IL-24, makes interpretation of these studies all the more complicated. Next to this, the role of IL-22BP might also be important in regulating IL-22 function, but it is unclear whether binding of IL-22 to IL-22BP has any effect on the microbiota-regulating properties of IL-22. Investigating changes in microbial composition in IL-22–/– animal models from birth or start of microbial environment exposure might shed more light on the establishment of beneficial microbial communities in the presence or absence of IL-22.

## Conclusion and Future Perspectives

**FIGURE 1:**
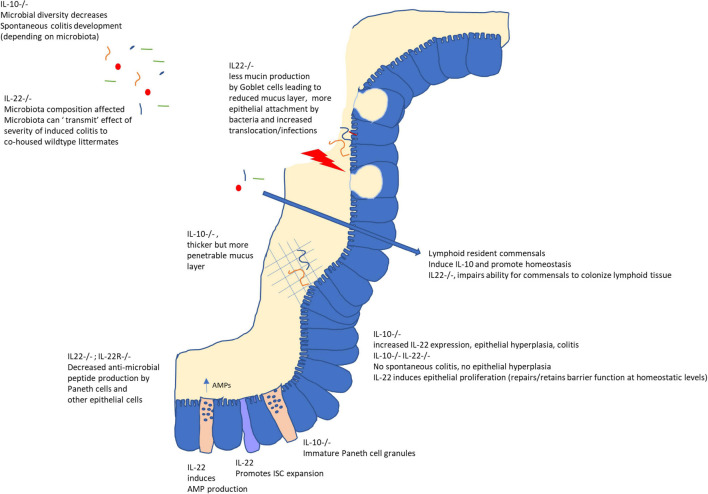
Role of IL-10 and IL-22 on the intestinal mucosal barrier and microbiota. While IL-10 is important to maintain homeostasis in the gut, IL-22 plays a dual role. In the absence of inflammation or infection, IL-22 signaling is important for epithelial proliferation, anti-microbial peptide (AMP) secretion and mucus secretion by Goblet cells as well as possibly allowing (IL-10-inducing) colonization of lymphoid tissue by selected microbes. However, in the case of infection or inflammation, increased IL-22 augments inflammatory processes as evidenced by its role in colitis in IL-10–/– mice and its requirement to, for example, combat *C. rodentium* infections. Interestingly, microbiota from IL22–/– mice could confer increased severity of chemically induced colitis to wildtype littermates, indicating these effects on the host (AMPs, mucin secretion) might also lead to an altered microbial composition.

As has become clear from recent advances in our understanding of host microbe interactions, the interplay between the environment, host and colonizing microbes involves complex and dynamic processes. In Figure 1 a schematic overview of IL-10 and IL-22 microbiota modifying effects is given. Early in life, bacterial species colonize the host. The host subsequently mounts both inflammatory and regulatory responses, in turn influencing the microbial composition. IL-22 has been convincingly shown to affect the microbiota composition and appears to create an environment that allows certain microbes to get the competitive advantage and even colonize host lymphoid tissue (resulting in IL-10 production). The exact mechanisms by which IL-22 enables some bacteria to colonize lymphoid tissues, whereas on the other hand is necessary to prevent epithelial-attachment via antimicrobial peptide induction and induction of mucus production, is currently unclear. For IL-10 the microbiota modulating potential is not so clear. The difficulty in comparing the different studies stems from the variation in co-housing and the use of littermates, as well as the timing of the experiments. IL-10–/– mice develop enterocolitis over time, making it difficult to address whether dysbiosis precedes or follows the inflammatory process. Detailed investigation into the first neonatal colonization processes might shed some light on the role of microbial induction of these regulatory cytokines (and cell types induced), as well as the effect of the induction of these regulatory cytokines on shaping the microbial community. This also holds true for the use of germfree models. Most studies compare germfree mice with wildtype mice at adult age. Likewise, when colonization effects are studied, mostly adult germfree mice are used, which might obscure those processes that are limited to (or play a bigger role) in early life. Although germfree mice have not experienced microbial colonization (and in that sense are naive) this does not mean that several other developmental processes are naive in their ability to respond to environmental stimuli. There might be a specific developmental window (‘a critical period in development in which an organism’s phenotype may be influenced by intrinsic and extrinsic factors,’ Burggren and Mueller, 2015) by which certain host processes can be influenced by the microbiota. Indeed, by comparing transcriptional profiles of intestinal regions of conventionalized (at adult age) and conventionally raised (from birth) germfree mice it was shown that the gene signature that distinguishes conventionally raised from adult conventionalized mice was region-specific as well as age-dependent (El Aidy et al., 2013b). Therefore, succession of microbial species (from pioneers to stable, yet dynamic, microbiota) in interaction with the host, might also influence host immunity in early life, while not being apparent at later stages. A deeper understanding on which immune cells and pathways can only be influenced during this window of opportunity early in life, and those that can be modified throughout life might help develop better targeted dietary (prebiotic) or probiotic intervention studies.

The processes under homeostatic conditions (more the focus of this review) might be very different from those in the context of inflammation. It has been reported for example, that IL-10–/– mice exhibited enhanced IL-22 responses toward *Clostridium difficile* infection (Cribas et al., 2021). Moreover, recently it was shown that spontaneous colitis in IL-10–/– mice is dependent on IL-22; IL-10–/–IL22–/– mice did not display spontaneous colitis (Gunasekera et al., 2020). IL10–/–IL-22–/– mice showed enhanced frequencies of Th17 cells, just like IL-10–/– mice, while microbial diversity in IL-10–/–IL-22–/– mice was restored (compared to IL10–/– mice) and no epithelial hyperplasia nor increased RegIIIγ was seen (Gunasekera et al., 2020).

Furthermore, IL-10 and IL-22 responses are shown to be dynamic and probably dependent on the location within the intestines and the life stage of the host. Indeed, it has been shown that in the ileum of wildtype mice ILC3 IL-22 expression increased until 4 weeks of life after which levels declined. This decrease after 4 weeks of age was not shown in Rag1–/– mice (lacking adaptive immune cells). Further investigation showed that both regulatory T cells and Th17 cells could diminish ILC3 activation by decreasing IL-23 production or regulation of the bacterial burden respectively (Mao et al., 2018). Thus it seems that early in life ILC3s shape the murine ileal microbiota through IL-23 and IL-22 dependent mechanisms, which is dampened with the development of adaptive immunity, when other mechanisms of host–microbiota cross-talk take over (Mao et al., 2018).

Differentiating between innate and adaptive sources of IL-10 and IL-22 is a challenge. Here, the zebrafish model might offer a solution. Since zebrafish develop *ex utero*, the early development of the immune system can be studied *in vivo* in a live animal using transgenic reporter zebrafish. Innate immunity develops from 2 dpf and adaptive immunity develops from 10 dpf onward (at this time CD4 + lymphocytes have been shown to leave the thymus). IL-22 and IL-10 seem to be conserved, although their regulation may differ (Costa et al., 2013; Siupka et al., 2014; Piazzon et al., 2016).

Furthermore, although most studies have focused on bacterial species interacting with the host, we now know that fungi (including yeasts), viruses/bacteriophages and protozoa should also be taken into account. Interestingly, viruses express IL-10 mimics influencing host responses (for review: Piazzon et al., 2016). Analysis of this intricate interplay between members of different kingdoms in the developing host is still in its infancy and predicts an exciting and challenging future for researchers. Focusing on the early life window and understanding possible long-term effects of microbial colonization on host immune development and general host health might enable smart targeted therapies to prevent chronic diseases later in life.

## Author Contributions

EK wrote the manuscript and made the table. MK wrote and edited the manuscript. SB wrote and edited the manuscript, made the figure, and secured the funding. All the authors contributed to the article and approved the submitted version.

## Conflict of Interest

The authors declare that the research was conducted in the absence of any commercial or financial relationships that could be construed as a potential conflict of interest.

## Publisher’s Note

All claims expressed in this article are solely those of the authors and do not necessarily represent those of their affiliated organizations, or those of the publisher, the editors and the reviewers. Any product that may be evaluated in this article, or claim that may be made by its manufacturer, is not guaranteed or endorsed by the publisher.

## Footnotes

1
ensembl.org

## References

[B1] Al Nabhani Z. Dulauroy S. Marques R. Cousu C. Al Bounny S. Dejardin F. (2019). A weaning reaction to microbiota is required for resistance to immunopathologies in the adult. *Immunity* 50 1276–1288.e5. 10.1016/j.immuni.2019.02.014 30902637

[B2] Arthur J. C. Gharaibeh R. Z. Muhlbauer M. Perez-Chanona E. Uronis J. M. McCafferty J. (2014). Microbial genomic analysis reveals the essential role of inflammation in bacteria-induced colorectal cancer. *Nat. Commun.* 5:4724. 10.1038/ncomms5724 25182170 PMC4155410

[B3] Arthur J. C. Perez-Chanona E. Muhlbauer M. Tomkovich S. Uronis J. M. Fan T. J. (2012). Intestinal inflammation targets cancer-inducing activity of the microbiota. *Science* 338 120–123. 10.1126/science.1224820 22903521 PMC3645302

[B4] Backhed F. Roswall J. Peng Y. Feng Q. Jia H. Kovatcheva-Datchary P. (2015). Dynamics and stabilization of the human gut microbiome during the first year of life. *Cell Host Microbe* 17:852. 10.1016/j.chom.2015.05.012 26308884

[B5] Bando J. K. Gilfillan S. Di Luccia B. Fachi J. L. Secca C. Cella M. (2020). ILC2s are the predominant source of intestinal ILC-derived IL-10. *J. Exp. Med.* 217:e20191520. 10.1084/jem.20191520 31699824 PMC7041711

[B6] Behnsen J. Jellbauer S. Wong C. P. Edwards R. A. George M. D. Ouyang W. (2014). The cytokine IL-22 promotes pathogen colonization by suppressing related commensal bacteria. *Immunity* 40 262–273. 10.1016/j.immuni.2014.01.003 24508234 PMC3964146

[B7] Belkaid Y. Naik S. (2013). Compartmentalized and systemic control of tissue immunity by commensals. *Nat. Immunol.* 14 646–653. 10.1038/ni.2604 23778791 PMC3845005

[B8] Bergstrom A. Skov T. H. Bahl M. I. Roager H. M. Christensen L. B. Ejlerskov K. T. (2014). Establishment of intestinal microbiota during early life: a longitudinal, explorative study of a large cohort of Danish infants. *Appl. Environ. Microbiol.* 80 2889–2900. 10.1128/AEM.00342-14 24584251 PMC3993305

[B9] Berkowitz L. Pardo-Roa C. Ramirez G. Vallejos O. P. Sebastian V. P. Riedel C. A. (2019). The absence of interleukin 10 affects the morphology, differentiation, granule content and the production of cryptidin-4 in Paneth cells in mice. *PLoS One* 14:e0221618. 10.1371/journal.pone.0221618 31509557 PMC6738610

[B10] Bevins C. L. Salzman N. H. (2011). The potter’s wheel: the host’s role in sculpting its microbiota. *Cell. Mol. Life Sci.* 68 3675–3685. 10.1007/s00018-011-0830-3 21968920 PMC3222938

[B11] Boniface K. Guignouard E. Pedretti N. Garcia M. Delwail A. Bernard F. X. (2007). A role for T cell-derived interleukin 22 in psoriatic skin inflammation. *Clin. Exp. Immunol.* 150 407–415. 10.1111/j.1365-2249.2007.03511.x 17900301 PMC2219373

[B12] Bottiglione F. Dee C. T. Lea R. Zeef L. A. H. Badrock A. P. Wane M. (2020). Zebrafish IL-4-like cytokines and IL-10 suppress inflammation but only IL-10 is essential for gill homeostasis. *J. Immunol.* 205 994–1008. 10.4049/jimmunol.2000372 32641385 PMC7416321

[B13] Brugman S. (2016). The zebrafish as a model to study intestinal inflammation. *Dev. Comp. Immunol.* 64 82–92. 10.1016/j.dci.2016.02.020 26902932

[B14] Brugman S. Liu K. Y. Lindenbergh-Kortleve D. Samsom J. N. Furuta G. T. Renshaw S. A. (2009). Oxazolone-induced enterocolitis in zebrafish depends on the composition of the intestinal microbiota. *Gastroenterology* 137 1757–1767.e1. 10.1053/j.gastro.2009.07.069 19698716

[B15] Bry L. Falk P. G. Midtvedt T. Gordon J. I. (1996). A model of host-microbial interactions in an open mammalian ecosystem. *Science* 273 1380–1383. 10.1126/science.273.5280.1380 8703071

[B16] Büchler G. Wos-Oxley M. L. Smoczek A. Zschemisch N. H. Neumann D. Pieper D. H. (2012). Strain-specific colitis susceptibility in IL10-deficient mice depends on complex gut microbiota-host interactions. *Inflamm. Bowel Dis.* 18 943–954. 10.1002/ibd.21895 22238116

[B17] Burggren W. W. Mueller C. A. (2015). Developmental critical windows and sensitive periods as three-dimensional constructs in time and space. *Physiol. Biochem. Zool.* 88 91–102. 10.1086/679906 25730265

[B18] Burns A. R. Stephens W. Z. Stagaman K. Wong S. Rawls J. F. Guillemin K. (2016). Contribution of neutral processes to the assembly of gut microbial communities in the zebrafish over host development. *ISME J.* 10 655–664. 10.1038/ismej.2015.142 26296066 PMC4817674

[B19] Cella M. Fuchs A. Vermi W. Facchetti F. Otero K. Lennerz J. K. (2009). A human natural killer cell subset provides an innate source of IL-22 for mucosal immunity. *Nature* 457 722–725. 10.1038/nature07537 18978771 PMC3772687

[B20] Colonna M. (2009). Interleukin-22-producing natural killer cells and lymphoid tissue inducer-like cells in mucosal immunity. *Immunity* 31 15–23. 10.1016/j.immuni.2009.06.008 19604490

[B21] Corripio-Miyar Y. Zou J. Richmond H. Secombes C. J. (2009). Identification of interleukin-22 in gadoids and examination of its expression level in vaccinated fish. *Mol. Immunol.* 46 2098–2106. 10.1016/j.molimm.2009.01.024 19403174

[B22] Costa M. M. Saraceni P. R. Forn-Cuni G. Dios S. Romero A. Figueras A. (2013). IL-22 is a key player in the regulation of inflammation in fish and involves innate immune cells and PI3K signaling. *Dev. Comp. Immunol.* 41 746–755. 10.1016/j.dci.2013.08.021 23999050

[B23] Cribas E. S. Denny J. E. Maslanka J. R. Abt M. C. (2021). Loss of Interleukin-10 (IL-10) Signaling Promotes IL-22-dependent host defenses against acute *Clostridioides difficile* infection. *Infect. Immun.* 89:e00730-20. 10.1128/IAI.00730-20 33649048 PMC8091099

[B24] Dee C. T. Nagaraju R. T. Athanasiadis E. I. Gray C. Fernandez Del Ama L. Johnston S. A. (2016). CD4-transgenic zebrafish reveal tissue-resident Th2- and regulatory T cell-like populations and diverse mononuclear phagocytes. *J. Immunol.* 197 3520–3530. 10.4049/jimmunol.1600959 27694495 PMC5073357

[B25] D’Hoe K. Vet S. Faust K. Moens F. Falony G. Gonze D. (2018). Integrated culturing, modeling and transcriptomics uncovers complex interactions and emergent behavior in a three-species synthetic gut community. *eLife* 7:e37090. 10.7554/eLife.37090 30322445 PMC6237439

[B26] Dieleman L. A. Arends A. Tonkonogy S. L. Goerres M. S. Craft D. W. Grenther W. (2000). *Helicobacter* hepaticus does not induce or potentiate colitis in interleukin-10-deficient mice. *Infect. Immun.* 68 5107–5113. 10.1128/IAI.68.9.5107-5113.2000 10948132 PMC101749

[B27] Donnelly R. P. Dickensheets H. Finbloom D. S. (1999). The interleukin-10 signal transduction pathway and regulation of gene expression in mononuclear phagocytes. *J. Interferon Cytokine Res.* 19 563–573. 10.1089/107999099313695 10433356

[B28] Driessler F. Venstrom K. Sabat R. Asadullah K. Schottelius A. J. (2004). Molecular mechanisms of interleukin-10-mediated inhibition of NF-kappaB activity: a role for p50. *Clin. Exp. Immunol.* 135 64–73. 10.1111/j.1365-2249.2004.02342.x 14678266 PMC1808913

[B29] Dudakov J. A. Hanash A. M. van den Brink M. R. (2015). Interleukin-22: immunobiology and pathology. *Annu. Rev. Immunol.* 33 747–785. 10.1146/annurev-immunol-032414-112123 25706098 PMC4407497

[B30] Dumoutier L. Van Roost E. Ameye G. Michaux L. Renauld J. C. (2000). IL-TIF/IL-22: genomic organization and mapping of the human and mouse genes. *Genes Immun.* 1 488–494. 10.1038/sj.gene.6363716 11197690

[B31] Egerton S. Culloty S. Whooley J. Stanton C. Ross R. P. (2018). The gut microbiota of marine fish. *Front. Microbiol.* 9:873. 10.3389/fmicb.2018.00873 29780377 PMC5946678

[B32] El Aidy S. Derrien M. Aardema R. Hooiveld G. Richards S. E. Dane A. (2014). Transient inflammatory-like state and microbial dysbiosis are pivotal in establishment of mucosal homeostasis during colonisation of germ-free mice. *Benef. Microbes* 5 67–77. 10.3920/Bm2013.0018 24322881

[B33] El Aidy S. Derrien M. Merrifield C. A. Levenez F. Dore J. Boekschoten M. V. (2013a). Gut bacteria-host metabolic interplay during conventionalisation of the mouse germfree colon. *ISME J.* 7 743–755. 10.1038/ismej.2012.142 23178667 PMC3603396

[B34] El Aidy S. Hooiveld G. Tremaroli V. Backhed F. Kleerebezem M. (2013b). The gut microbiota and mucosal homeostasis: Colonized at birth or at adulthood, does it matter? *Gut Microbes* 4 118–124. 10.4161/gmic.23362 23333858 PMC3595071

[B35] El Aidy S. Merrifield C. A. Derrien M. van Baarlen P. Hooiveld G. Levenez F. (2013c). The gut microbiota elicits a profound metabolic reorientation in the mouse jejunal mucosa during conventionalisation. *Gut* 62 1306–1314. 10.1136/gutjnl-2011-301955 22722618

[B36] El Aidy S. van Baarlen P. Derrien M. Lindenbergh-Kortleve D. J. Hooiveld G. Levenez F. (2012). Temporal and spatial interplay of microbiota and intestinal mucosa drive establishment of immune homeostasis in conventionalized mice. *Mucosal Immunol.* 5 567–579. 10.1038/mi.2012.32 22617837

[B37] Engelhardt K. R. Grimbacher B. (2014). IL-10 in humans: lessons from the gut, IL-10/IL-10 receptor deficiencies, and IL-10 polymorphisms. *Curr. Top. Microbiol. Immunol.* 380 1–18. 10.1007/978-3-662-43492-5_1 25004811

[B38] Eyerich S. Eyerich K. Pennino D. Carbone T. Nasorri F. Pallotta S. (2009). Th22 cells represent a distinct human T cell subset involved in epidermal immunity and remodeling. *J. Clin. Invest.* 119 3573–3585. 10.1172/JCI40202 19920355 PMC2786807

[B39] Fatkhullina A. R. Peshkova I. O. Dzutsev A. Aghayev T. McCulloch J. A. Thovarai V. (2018). An interleukin-23-Interleukin-22 Axis regulates intestinal microbial homeostasis to protect from diet-induced atherosclerosis. *Immunity* 49 943–957.e9. 10.1016/j.immuni.2018.09.011 30389414 PMC6257980

[B40] Fillatreau S. O’Garra A. (eds) (2014). “Interleukin 10 in health and disease,” in *Current Topics in Microbiology and Immunology* (Berlin: Springer-Verlag). 10.1007/978-3-662-43492-5

[B41] Fung T. C. Bessman N. J. Hepworth M. R. Kumar N. Shibata N. Kobuley D. (2016). Lymphoid-tissue-resident commensal bacteria promote members of the IL-10 cytokine family to establish Mutualism. *Immunity* 44 634–646. 10.1016/j.immuni.2016.02.019 26982365 PMC4845739

[B42] Garrett W. S. Lord G. M. Punit S. Lugo-Villarino G. Mazmanian S. K. Ito S. (2007). Communicable ulcerative colitis induced by T-bet deficiency in the innate immune system. *Cell* 131 33–45. 10.1016/j.cell.2007.08.017 17923086 PMC2169385

[B43] Gaudino S. J. Beaupre M. Lin X. Joshi P. Rathi S. McLaughlin P. A. (2021). IL-22 receptor signaling in Paneth cells is critical for their maturation, microbiota colonization, Th17-related immune responses, and anti-*Salmonella* immunity. *Mucosal Immunol.* 14 389–401. 10.1038/s41385-020-00348-5 33060802 PMC7946635

[B44] Glocker E. O. Kotlarz D. Boztug K. Gertz E. M. Schaffer A. A. Noyan F. (2009). Inflammatory bowel disease and mutations affecting the interleukin-10 receptor. *N. Engl. J. Med.* 361 2033–2045. 10.1056/NEJMoa0907206 19890111 PMC2787406

[B45] Gunasekera D. C. Ma J. Vacharathit V. Shah P. Ramakrishnan A. Uprety P. (2020). The development of colitis in Il10(-/-) mice is dependent on IL-22. *Mucosal Immunol.* 13 493–506. 10.1038/s41385-019-0252-3 31932715 PMC7566780

[B46] Guo W. Luo C. Wang C. Wang X. Wang X. Gao X. D. (2014). Suppression of human and mouse Th17 differentiation and autoimmunity by an endogenous Interleukin 23 receptor cytokine-binding homology region. *Int. J. Biochem. Cell Biol.* 55 304–310. 10.1016/j.biocel.2014.09.019 25263529

[B47] Hansson M. Silverpil E. Linden A. Glader P. (2013). Interleukin-22 produced by alveolar macrophages during activation of the innate immune response. *Inflamm. Res.* 62 561–569. 10.1007/s00011-013-0608-1 23474919

[B48] Harjula S. E. Ojanen M. J. T. Taavitsainen S. Nykter M. Ramet M. (2018). Interleukin 10 mutant zebrafish have an enhanced interferon gamma response and improved survival against a Mycobacterium marinum infection. *Sci. Rep.* 8:10360. 10.1038/s41598-018-28511-w 29985419 PMC6037744

[B49] Hart M. L. Ericsson A. C. Franklin C. L. (2017). Differing complex microbiota alter disease severity of the IL-10(-/-) Mouse Model of inflammatory bowel disease. *Front. Microbiol.* 8:792. 10.3389/fmicb.2017.00792 28553262 PMC5425584

[B50] Hernandez P. P. Strzelecka P. M. Athanasiadis E. I. Hall D. Robalo A. F. Collins C. M. (2018). Single-cell transcriptional analysis reveals ILC-like cells in zebrafish. *Sci. Immunol.* 3:eaau5265. 10.1126/sciimmunol.aau5265 30446505 PMC6258902

[B51] Hooper L. V. Littman D. R. Macpherson A. J. (2012). Interactions between the microbiota and the immune system. *Science* 336 1268–1273. 10.1126/science.1223490 22674334 PMC4420145

[B52] Hooper L. V. Xu J. Falk P. G. Midtvedt T. Gordon J. I. (1999). A molecular sensor that allows a gut commensal to control its nutrient foundation in a competitive ecosystem. *Proc. Natl. Acad. Sci. U.S.A.* 96 9833–9838. 10.1073/pnas.96.17.9833 10449780 PMC22296

[B53] Huo H. J. Chen S. N. Li L. Laghari Z. A. Li N. Nie P. (2019). Functional characterization of interleukin (IL)-22 and its inhibitor, IL-22 binding protein (IL-22BP) in Mandarin fish, *Siniperca chuatsi*. *Dev. Comp. Immunol.* 97 88–97. 10.1016/j.dci.2019.03.007 30902735

[B54] Huus K. E. Petersen C. Finlay B. B. (2021). Diversity and dynamism of IgA-microbiota interactions. *Nat. Rev. Immunol.* 21 514–525. 10.1038/s41577-021-00506-1 33568782

[B55] Igawa D. Sakai M. Savan R. (2006). An unexpected discovery of two interferon gamma-like genes along with interleukin (IL)-22 and -26 from teleost: IL-22 and -26 genes have been described for the first time outside mammals. *Mol. Immunol.* 43 999–1009. 10.1016/j.molimm.2005.05.009 16005068

[B56] Ivanov I. I. Atarashi K. Manel N. Brodie E. L. Shima T. Karaoz U. (2009). Induction of intestinal Th17 cells by segmented filamentous bacteria. *Cell* 139 485–498. 10.1016/j.cell.2009.09.033 19836068 PMC2796826

[B57] Izcue A. Coombes J. L. Powrie F. (2006). Regulatory T cells suppress systemic and mucosal immune activation to control intestinal inflammation. *Immunol. Rev.* 212 256–271. 10.1111/j.0105-2896.2006.00423.x 16903919

[B58] Izcue A. Powrie F. (2008). Special regulatory T-cell review: regulatory T cells and the intestinal tract–patrolling the frontier. *Immunology* 123 6–10. 10.1111/j.1365-2567.2007.02778.x 18154611 PMC2433286

[B59] Johansson M. E. Gustafsson J. K. Holmen-Larsson J. Jabbar K. S. Xia L. Xu H. (2014). Bacteria penetrate the normally impenetrable inner colon mucus layer in both murine colitis models and patients with ulcerative colitis. *Gut* 63 281–291. 10.1136/gutjnl-2012-303207 23426893 PMC3740207

[B60] Kasama T. Strieter R. M. Lukacs N. W. Burdick M. D. Kunkel S. L. (1994). Regulation of neutrophil-derived chemokine expression by IL-10. *J. Immunol.* 152 3559–3569. 8144935

[B61] Keir M. Yi Y. Lu T. Ghilardi N. (2020). The role of IL-22 in intestinal health and disease. *J. Exp. Med.* 217:e20192195. 10.1084/jem.20192195 32997932 PMC7062536

[B62] Kern L. Abdeen S. K. Kolodziejczyk A. A. Elinav E. (2021). Commensal inter-bacterial interactions shaping the microbiota. *Curr. Opin. Microbiol.* 63 158–171. 10.1016/j.mib.2021.07.011 34365152

[B63] Kim S. C. Tonkonogy S. L. Albright C. A. Tsang J. Balish E. J. Braun J. (2005). Variable phenotypes of enterocolitis in interleukin 10-deficient mice monoassociated with two different commensal bacteria. *Gastroenterology* 128 891–906. 10.1053/j.gastro.2005.02.009 15825073

[B64] Kimmel C. B. Ballard W. M. Kimmel S. R. Ullmann B. Schilling T. F. (1995). Stages of Embryonic Development of the Zebrafish. *Dev. Dyn.* 203 255–310. 10.1002/aja.1002030302 8589427

[B65] Kotenko S. V. Izotova L. S. Mirochnitchenko O. V. Esterova E. Dickensheets H. Donnelly R. P. (2001a). Identification of the functional interleukin-22 (IL-22) receptor complex: the IL-10R2 chain (IL-10Rbeta) is a common chain of both the IL-10 and IL-22 (IL-10-related T cell-derived inducible factor, IL-TIF) receptor complexes. *J. Biol. Chem.* 276 2725–2732. 10.1074/jbc.M007837200 11035029

[B66] Kotenko S. V. Izotova L. S. Mirochnitchenko O. V. Esterova E. Dickensheets H. Donnelly R. P. (2001b). Identification, cloning, and characterization of a novel soluble receptor that binds IL-22 and neutralizes its activity. *J. Immunol.* 166 7096–7103. 10.4049/jimmunol.166.12.7096 11390454

[B67] Kotlarz D. Beier R. Murugan D. Diestelhorst J. Jensen O. Boztug K. (2012). Loss of interleukin-10 signaling and infantile inflammatory bowel disease: implications for diagnosis and therapy. *Gastroenterology* 143 347–355. 10.1053/j.gastro.2012.04.045 22549091

[B68] Kühn R. Lohler J. Rennick D. Rajewsky K. Muller W. (1993). Interleukin-10-deficient mice develop chronic enterocolitis. *Cell* 75 263–274. 10.1016/0092-8674(93)80068-p 8402911

[B69] Kunisawa J. Gohda M. Hashimoto E. Ishikawa I. Higuchi M. Suzuki Y. (2013). Microbe-dependent CD11b+ IgA+ plasma cells mediate robust early-phase intestinal IgA responses in mice. *Nat. Commun.* 4:1772. 10.1038/ncomms2718 23612313 PMC3644083

[B70] Lam S. H. Chua H. L. Gong Z. Lam T. J. Sin Y. M. (2004). Development and maturation of the immune system in zebrafish, Danio rerio: a gene expression profiling, in situ hybridization and immunological study. *Dev. Comp. Immunol.* 28 9–28. 10.1016/s0145-305x(03)00103-4 12962979

[B71] Laukens D. Brinkman B. M. Raes J. De Vos M. Vandenabeele P. (2016). Heterogeneity of the gut microbiome in mice: guidelines for optimizing experimental design. *FEMS Microbiol. Rev.* 40 117–132. 10.1093/femsre/fuv036 26323480 PMC4703068

[B72] Lee S. M. Donaldson G. P. Mikulski Z. Boyajian S. Ley K. Mazmanian S. K. (2013). Bacterial colonization factors control specificity and stability of the gut microbiota. *Nature* 501 426–429. 10.1038/nature12447 23955152 PMC3893107

[B73] Levraud J. P. Boudinot P. Colin I. Benmansour A. Peyrieras N. Herbomel P. (2007). Identification of the zebrafish IFN receptor: implications for the origin of the vertebrate IFN system. *J. Immunol.* 178 4385–4394. 10.4049/jimmunol.178.7.4385 17371995

[B74] Li J. Tomkinson K. N. Tan X. Y. Wu P. Yan G. Spaulding V. (2004). Temporal associations between interleukin 22 and the extracellular domains of IL-22R and IL-10R2. *Int. Immunopharmacol.* 4 693–708. 10.1016/j.intimp.2004.01.010 15120653

[B75] Li Y. Li Y. Cao X. Jin X. Jin T. (2017). Pattern recognition receptors in zebrafish provide functional and evolutionary insight into innate immune signaling pathways. *Cell. Mol. Immunol.* 14 80–89. 10.1038/cmi.2016.50 27721456 PMC5214946

[B76] Liang S. C. Tan X. Y. Luxenberg D. P. Karim R. Dunussi-Joannopoulos K. Collins M. (2006). Interleukin (IL)-22 and IL-17 are coexpressed by Th17 cells and cooperatively enhance expression of antimicrobial peptides. *J. Exp. Med.* 203 2271–2279. 10.1084/jem.20061308 16982811 PMC2118116

[B77] Lindemans C. A. Calafiore M. Mertelsmann A. M. O’Connor M. H. Dudakov J. A. Jenq R. R. (2015). Interleukin-22 promotes intestinal-stem-cell-mediated epithelial regeneration. *Nature* 528 560–564. 10.1038/nature16460 26649819 PMC4720437

[B78] Liu Y. de Bruijn I. Jack A. L. Drynan K. van den Berg A. H. Thoen E. (2014). Deciphering microbial landscapes of fish eggs to mitigate emerging diseases. *ISME J.* 8 2002–2014. 10.1038/ismej.2014.44 24671087 PMC4184010

[B79] Lysenko E. S. Ratner A. J. Nelson A. L. Weiser J. N. (2005). The role of innate immune responses in the outcome of interspecies competition for colonization of mucosal surfaces. *PLoS Pathog.* 1:e1. 10.1371/journal.ppat.0010001 16201010 PMC1238736

[B80] Maharshak N. Packey C. D. Ellermann M. Manick S. Siddle J. P. Huh E. Y. (2013). Altered enteric microbiota ecology in interleukin 10-deficient mice during development and progression of intestinal inflammation. *Gut Microbes* 4 316–324. 10.4161/gmic.25486 23822920 PMC3744516

[B81] Mao K. Baptista A. P. Tamoutounour S. Zhuang L. Bouladoux N. Martins A. J. (2018). Innate and adaptive lymphocytes sequentially shape the gut microbiota and lipid metabolism. *Nature* 554 255–259. 10.1038/nature25437 29364878

[B82] Marcobal A. Barboza M. Sonnenburg E. D. Pudlo N. Martens E. C. Desai P. (2011). *Bacteroides* in the infant gut consume milk oligosaccharides via mucus-utilization pathways. *Cell Host Microbe* 10 507–514. 10.1016/j.chom.2011.10.007 22036470 PMC3227561

[B83] Mishima Y. Oka A. Liu B. Herzog J. W. Eun C. S. Fan T. J. (2019). Microbiota maintain colonic homeostasis by activating TLR2/MyD88/PI3K signaling in IL-10-producing regulatory B cells. *J. Clin. Invest.* 129 3702–3716. 10.1172/JCI93820 31211700 PMC6715367

[B84] Miyamoto C. Kojo S. Yamashita M. Moro K. Lacaud G. Shiroguchi K. (2019). Runx/Cbfbeta complexes protect group 2 innate lymphoid cells from exhausted-like hyporesponsiveness during allergic airway inflammation. *Nat. Commun.* 10:447. 10.1038/s41467-019-08365-0 30683858 PMC6347616

[B85] Monte M. M. Zou J. Wang T. Carrington A. Secombes C. J. (2011). Cloning, expression analysis and bioactivity studies of rainbow trout (*Oncorhynchus mykiss*) interleukin-22. *Cytokine* 55 62–73. 10.1016/j.cyto.2011.03.015 21514178

[B86] Munoz M. Eidenschenk C. Ota N. Wong K. Lohmann U. Kuhl A. A. (2015). Interleukin-22 induces interleukin-18 expression from epithelial cells during intestinal infection. *Immunity* 42 321–331. 10.1016/j.immuni.2015.01.011 25680273

[B87] Murai M. Turovskaya O. Kim G. Madan R. Karp C. L. Cheroutre H. (2009). Interleukin 10 acts on regulatory T cells to maintain expression of the transcription factor Foxp3 and suppressive function in mice with colitis. *Nat. Immunol.* 10 1178–1184. 10.1038/ni.1791 19783988 PMC2898179

[B88] Nagao-Kitamoto H. Leslie J. L. Kitamoto S. Jin C. Thomsson K. A. Gillilland M. G. (2020). Interleukin-22-mediated host glycosylation prevents *Clostridioides difficile* infection by modulating the metabolic activity of the gut microbiota. *Nat. Med.* 26 608–617. 10.1038/s41591-020-0764-0 32066975 PMC7160049

[B89] Neurath M. F. (2019). IL-23 in inflammatory bowel diseases and colon cancer. *Cytokine Growth Factor Rev.* 45 1–8. 10.1016/j.cytogfr.2018.12.002 30563755

[B90] Oehlers S. H. Flores M. V. Hall C. J. Crosier K. E. Crosier P. S. (2012). Retinoic acid suppresses intestinal mucus production and exacerbates experimental enterocolitis. *Dis. Model. Mech.* 5 457–467. 10.1242/dmm.009365 22563081 PMC3380709

[B91] Olszak T. Neves J. F. Dowds C. M. Baker K. Glickman J. Davidson N. O. (2014). Protective mucosal immunity mediated by epithelial CD1d and IL-10. *Nature* 509 497–502. 10.1038/nature13150 24717441 PMC4132962

[B92] Ouyang W. Rutz S. Crellin N. K. Valdez P. A. Hymowitz S. G. (2011). Regulation and functions of the IL-10 family of cytokines in inflammation and disease. *Annu. Rev. Immunol.* 29 71–109. 10.1146/annurev-immunol-031210-101312 21166540

[B93] Overstreet A. C. Ramer-Tait A. E. Suchodolski J. S. Hostetter J. M. Wang C. Jergens A. E. (2021). Temporal dynamics of chronic inflammation on the Cecal Microbiota in IL-10(-/-) Mice. *Front. Immunol.* 11:585431. 10.3389/fimmu.2020.585431 33664728 PMC7921487

[B94] Pannaraj P. S. Li F. Cerini C. Bender J. M. Yang S. Rollie A. (2017). Association between breast milk bacterial communities and establishment and development of the infant gut microbiome. *JAMA Pediatr.* 171 647–654. 10.1001/jamapediatrics.2017.0378 28492938 PMC5710346

[B95] Parks O. B. Pociask D. A. Hodzic Z. Kolls J. K. Good M. (2015). Interleukin-22 signaling in the regulation of intestinal health and disease. *Front. Cell Dev. Biol.* 3:85. 10.3389/fcell.2015.00085 26793707 PMC4710696

[B96] Perez-Munoz M. E. Arrieta M. C. Ramer-Tait A. E. Walter J. (2017). A critical assessment of the “sterile womb” and “in utero colonization” hypotheses: implications for research on the pioneer infant microbiome. *Microbiome* 5:48. 10.1186/s40168-017-0268-4 28454555 PMC5410102

[B97] Pham T. A. Clare S. Goulding D. Arasteh J. M. Stares M. D. Browne H. P. (2014). Epithelial IL-22RA1-mediated fucosylation promotes intestinal colonization resistance to an opportunistic pathogen. *Cell Host Microbe* 16 504–516. 10.1016/j.chom.2014.08.017 25263220 PMC4190086

[B98] Piazzon M. C. Lutfalla G. Forlenza M. (2016). IL10, a tale of an evolutionarily conserved cytokine across vertebrates. *Crit. Rev. Immunol.* 36 99–129. 10.1615/CritRevImmunol.2016017480 27910763

[B99] Qiu J. Guo X. Chen Z. M. He L. Sonnenberg G. F. Artis D. (2013). Group 3 innate lymphoid cells inhibit T-cell-mediated intestinal inflammation through aryl hydrocarbon receptor signaling and regulation of microflora. *Immunity* 39 386–399. 10.1016/j.immuni.2013.08.002 23954130 PMC3884586

[B100] Radaeva S. Sun R. Pan H. N. Hong F. Gao B. (2004). Interleukin 22 (IL-22) plays a protective role in T cell-mediated murine hepatitis: IL-22 is a survival factor for hepatocytes via STAT3 activation. *Hepatology* 39 1332–1342. 10.1002/hep.20184 15122762

[B101] Rawls J. F. Mahowald M. A. Ley R. E. Gordon J. I. (2006). Reciprocal gut microbiota transplants from zebrafish and mice to germ-free recipients reveal host habitat selection. *Cell* 127 423–433. 10.1016/j.cell.2006.08.043 17055441 PMC4839475

[B102] Rawls J. F. Samuel B. S. Gordon J. I. (2004). Gnotobiotic zebrafish reveal evolutionarily conserved responses to the gut microbiota. *Proc. Natl. Acad. Sci. U.S.A.* 101 4596–4601. 10.1073/pnas.0400706101 15070763 PMC384792

[B103] Redhu N. S. Bakthavatchalu V. Conaway E. A. Shouval D. S. Tsou A. Goettel J. A. (2017). Macrophage dysfunction initiates colitis during weaning of infant mice lacking the interleukin-10 receptor. *eLife* 6:e27652. 10.7554/eLife.27652 28678006 PMC5531923

[B104] Rendueles O. Ferrieres L. Fretaud M. Begaud E. Herbomel P. Levraud J. P. (2012). A new zebrafish model of oro-intestinal pathogen colonization reveals a key role for adhesion in protection by probiotic bacteria. *PLoS Pathog.* 8:e1002815. 10.1371/journal.ppat.1002815 22911651 PMC3406073

[B105] Renz H. Brandtzaeg P. Hornef M. (2011). The impact of perinatal immune development on mucosal homeostasis and chronic inflammation. *Nat. Rev. Immunol.* 12 9–23. 10.1038/nri3112 22158411

[B106] Riley J. K. Takeda K. Akira S. Schreiber R. D. (1999). Interleukin-10 receptor signaling through the JAK-STAT pathway. Requirement for two distinct receptor-derived signals for anti-inflammatory action. *J. Biol. Chem.* 274 16513–16521. 10.1074/jbc.274.23.16513 10347215

[B107] Rivollier A. He J. Kole A. Valatas V. Kelsall B. L. (2012). Inflammation switches the differentiation program of Ly6Chi monocytes from antiinflammatory macrophages to inflammatory dendritic cells in the colon. *J. Exp. Med.* 209 139–155. 10.1084/jem.20101387 22231304 PMC3260867

[B108] Robinson C. D. Klein H. S. Murphy K. D. Parthasarathy R. Guillemin K. Bohannan B. J. M. (2018). Experimental bacterial adaptation to the zebrafish gut reveals a primary role for immigration. *PLoS Biol.* 16:e2006893. 10.1371/journal.pbio.2006893 30532251 PMC6301714

[B109] Roeselers G. Mittge E. K. Stephens W. Z. Parichy D. M. Cavanaugh C. M. Guillemin K. (2011). Evidence for a core gut microbiota in the zebrafish. *ISME J.* 5 1595–1608. 10.1038/ismej.2011.38 21472014 PMC3176511

[B110] Round J. L. Lee S. M. Li J. Tran G. Jabri B. Chatila T. A. (2011). The Toll-like receptor 2 pathway establishes colonization by a commensal of the human microbiota. *Science* 332 974–977. 10.1126/science.1206095 21512004 PMC3164325

[B111] Rutz S. Ouyang W. (2011). Regulation of interleukin-10 and interleukin-22 expression in T helper cells. *Curr. Opin. Immunol.* 23 605–612. 10.1016/j.coi.2011.07.018 21862302

[B112] Samuel C. Eisen J. S. Farmer S. F. Guillemin K. J. Kent M. L. Sanders G. E. (eds) (2019). “The Zebrafish in biomedical research,” in *American College of Laboratory Animal Medicine*, 1st Edn, (Cambridge, MA: Academic Press). 10.1016/B978-0-12-812431-4.05001-6

[B113] Saraiva M. O’Garra A. (2010). The regulation of IL-10 production by immune cells. *Nat. Rev. Immunol.* 10 170–181. 10.1038/nri2711 20154735

[B114] Scales B. S. Dickson R. P. Huffnagle G. B. (2016). A tale of two sites: how inflammation can reshape the microbiomes of the gut and lungs. *J. Leukoc. Biol.* 100 943–950. 10.1189/jlb.3MR0316-106R 27365534 PMC5069096

[B115] Schmechel S. Konrad A. Diegelmann J. Glas J. Wetzke M. Paschos E. (2008). Linking genetic susceptibility to Crohn’s disease with Th17 cell function: IL-22 serum levels are increased in Crohn’s disease and correlate with disease activity and IL23R genotype status. *Inflamm. Bowel Dis.* 14 204–212. 10.1002/ibd.20315 18022867

[B116] Seehus C. R. Kadavallore A. Torre B. Yeckes A. R. Wang Y. Tang J. (2017). Alternative activation generates IL-10 producing type 2 innate lymphoid cells. *Nat. Commun.* 8:1900. 10.1038/s41467-017-02023-z 29196657 PMC5711851

[B117] Sellon R. K. Tonkonogy S. Schultz M. Dieleman L. A. Grenther W. Balish E. (1998). Resident enteric bacteria are necessary for development of spontaneous colitis and immune system activation in interleukin-10-deficient mice. *Infect. Immun.* 66 5224–5231. 10.1128/IAI.66.11.5224-5231.1998 9784526 PMC108652

[B118] Shohan M. Dehghani R. Khodadadi A. Dehnavi S. Ahmadi R. Joudaki N. (2020). Interleukin-22 and intestinal homeostasis: Protective or destructive? *IUBMB Life* 72 1585–1602. 10.1002/iub.2295 32365282

[B119] Shouval D. S. Biswas A. Goettel J. A. McCann K. Conaway E. Redhu N. S. (2014). Interleukin-10 receptor signaling in innate immune cells regulates mucosal immune tolerance and anti-inflammatory macrophage function. *Immunity* 40 706–719. 10.1016/j.immuni.2014.03.011 24792912 PMC4513358

[B120] Siupka P. Hamming O. J. Fretaud M. Luftalla G. Levraud J. P. Hartmann R. (2014). The crystal structure of zebrafish IL-22 reveals an evolutionary, conserved structure highly similar to that of human IL-22. *Genes Immun.* 15 293–302. 10.1038/gene.2014.18 24833303

[B121] Sonnenberg G. F. Monticelli L. A. Alenghat T. Fung T. C. Hutnick N. A. Kunisawa J. (2012). Innate lymphoid cells promote anatomical containment of lymphoid-resident commensal bacteria. *Science* 336 1321–1325. 10.1126/science.1222551 22674331 PMC3659421

[B122] Stein C. Caccamo M. Laird G. Leptin M. (2007). Conservation and divergence of gene families encoding components of innate immune response systems in zebrafish. *Genome Biol.* 8:R251. 10.1186/gb-2007-8-11-r251 18039395 PMC2258186

[B123] Stephens W. Z. Burns A. R. Stagaman K. Wong S. Rawls J. F. Guillemin K. (2016). The composition of the zebrafish intestinal microbial community varies across development. *ISME J.* 10 644–654. 10.1038/ismej.2015.140 26339860 PMC4817687

[B124] Stevens E. J. Bates K. A. King K. C. (2021). Host microbiota can facilitate pathogen infection. *PLoS Pathog.* 17:e1009514. 10.1371/journal.ppat.1009514 33984069 PMC8118302

[B125] Stewart C. J. Ajami N. J. O’Brien J. L. Hutchinson D. S. Smith D. P. Wong M. C. (2018). Temporal development of the gut microbiome in early childhood from the TEDDY study. *Nature* 562 583–588. 10.1038/s41586-018-0617-x 30356187 PMC6415775

[B126] Sugimoto K. Ogawa A. Mizoguchi E. Shimomura Y. Andoh A. Bhan A. K. (2008). IL-22 ameliorates intestinal inflammation in a mouse model of ulcerative colitis. *J. Clin. Invest.* 118 534–544. 10.1172/JCI33194 18172556 PMC2157567

[B127] Sullam K. E. Essinger S. D. Lozupone C. A. O’Connor M. P. Rosen G. L. Knight R. (2012). Environmental and ecological factors that shape the gut bacterial communities of fish: a meta-analysis. *Mol. Ecol.* 21 3363–3378. 10.1111/j.1365-294X.2012.05552.x 22486918 PMC3882143

[B128] Sung J. Kim S. Cabatbat J. J. T. Jang S. Jin Y. S. Jung G. Y. (2017). Global metabolic interaction network of the human gut microbiota for context-specific community-scale analysis. *Nat. Commun.* 8:15393. 10.1038/ncomms15393 28585563 PMC5467172

[B129] Sydora B. C. Tavernini M. M. Doyle J. S. Fedorak R. N. (2005). Association with selected bacteria does not cause enterocolitis in IL-10 gene-deficient mice despite a systemic immune response. *Dig. Dis. Sci.* 50 905–913. 10.1007/s10620-005-2663-0 15906767

[B130] Thiennimitr P. Winter S. E. Winter M. G. Xavier M. N. Tolstikov V. Huseby D. L. (2011). Intestinal inflammation allows *Salmonella* to use ethanolamine to compete with the microbiota. *Proc. Natl. Acad. Sci. U.S.A.* 108 17480–17485. 10.1073/pnas.1107857108 21969563 PMC3198331

[B131] Timmerman H. M. Rutten N. Boekhorst J. Saulnier D. M. Kortman G. A. M. Contractor N. (2017). Intestinal colonisation patterns in breastfed and formula-fed infants during the first 12 weeks of life reveal sequential microbiota signatures. *Sci. Rep.* 7:8327. 10.1038/s41598-017-08268-4 28827640 PMC5567133

[B132] Tsai P. Y. Zhang B. He W. Q. Zha J. M. Odenwald M. A. Singh G. (2017). IL-22 Upregulates epithelial claudin-2 to drive diarrhea and enteric pathogen clearance. *Cell Host Microbe* 21 671–681.e4. 10.1016/j.chom.2017.05.009 28618266 PMC5541253

[B133] Ueda Y. Kayama H. Jeon S. G. Kusu T. Isaka Y. Rakugi H. (2010). Commensal microbiota induce LPS hyporesponsiveness in colonic macrophages via the production of IL-10. *Int. Immunol.* 22 953–962. 10.1093/intimm/dxq449 21051439

[B134] Upadhyay V. Poroyko V. Kim T. J. Devkota S. Fu S. Liu D. (2012). Lymphotoxin regulates commensal responses to enable diet-induced obesity. *Nat. Immunol.* 13 947–953. 10.1038/ni.2403 22922363 PMC3718316

[B135] Wang J. Chen W. D. Wang Y. D. (2020). The relationship between gut microbiota and inflammatory diseases: the role of macrophages. *Front. Microbiol.* 11:1065. 10.3389/fmicb.2020.01065 32582063 PMC7296120

[B136] Wei H. Wang X. Zhang A. Du L. Zhou H. (2014). Identification of grass carp IL-10 receptor subunits: functional evidence for IL-10 signaling in teleost immunity. *Dev. Comp. Immunol.* 45 259–268. 10.1016/j.dci.2014.03.012 24690565

[B137] Wei H. X. Wang B. Li B. (2020). IL-10 and IL-22 in Mucosal immunity: driving protection and pathology. *Front. Immunol.* 11:1315. 10.3389/fimmu.2020.01315 32670290 PMC7332769

[B138] Willems F. Marchant A. Delville J. P. Gerard C. Delvaux A. Velu T. (1994). Interleukin-10 inhibits B7 and intercellular adhesion molecule-1 expression on human monocytes. *Eur. J. Immunol.* 24 1007–1009. 10.1002/eji.1830240435 7512027

[B139] Wolk K. Kunz S. Witte E. Friedrich M. Asadullah K. Sabat R. (2004). IL-22 increases the innate immunity of tissues. *Immunity* 21 241–254. 10.1016/j.immuni.2004.07.007 15308104

[B140] Wolk K. Witte E. Wallace E. Docke W. D. Kunz S. Asadullah K. (2006). IL-22 regulates the expression of genes responsible for antimicrobial defense, cellular differentiation, and mobility in keratinocytes: a potential role in psoriasis. *Eur. J. Immunol.* 36 1309–1323. 10.1002/eji.200535503 16619290

[B141] Xiao F. Zhu W. Yu Y. He Z. Wu B. Wang C. (2021). Host development overwhelms environmental dispersal in governing the ecological succession of zebrafish gut microbiota. *NPJ Biofilms Microbiomes* 7:5. 10.1038/s41522-020-00176-2 33469034 PMC7815754

[B142] Xie M. H. Aggarwal S. Ho W. H. Foster J. Zhang Z. Stinson J. (2000). Interleukin (IL)-22, a novel human cytokine that signals through the interferon receptor-related proteins CRF2-4 and IL-22R. *J. Biol. Chem.* 275 31335–31339. 10.1074/jbc.M005304200 10875937

[B143] Yang Y. Takeda A. Yoshimura T. Oshima Y. Sonoda K. H. Ishibashi T. (2013). IL-10 is significantly involved in HSP70-regulation of experimental subretinal fibrosis. *PLoS One* 8:e80288. 10.1371/journal.pone.0080288 24376495 PMC3869650

[B144] Yang Y. Wang J. Xu J. Liu Q. Wang Z. Zhu X. (2020). Characterization of IL-22 Bioactivity and IL-22-Positive Cells in Grass Carp *Ctenopharyngodon idella*. *Front. Immunol.* 11:586889. 10.3389/fimmu.2020.586889 33178219 PMC7593840

[B145] Yeste A. Mascanfroni I. D. Nadeau M. Burns E. J. Tukpah A. M. Santiago A. (2014). IL-21 induces IL-22 production in CD4+ T cells. *Nat. Commun.* 5:3753. 10.1038/ncomms4753 24796415 PMC4157605

[B146] Zenewicz L. A. Yin X. Wang G. Elinav E. Hao L. Zhao L. (2013). IL-22 deficiency alters colonic microbiota to be transmissible and colitogenic. *J. Immunol.* 190 5306–5312. 10.4049/jimmunol.1300016 23585682 PMC3646987

[B147] Zheng Y. Valdez P. A. Danilenko D. M. Hu Y. Sa S. M. Gong Q. (2008). Interleukin-22 mediates early host defense against attaching and effacing bacterial pathogens. *Nat. Med.* 14 282–289. 10.1038/nm1720 18264109

[B148] Zindl C. L. Lai J. F. Lee Y. K. Maynard C. L. Harbour S. N. Ouyang W. (2013). IL-22-producing neutrophils contribute to antimicrobial defense and restitution of colonic epithelial integrity during colitis. *Proc. Natl. Acad. Sci. U.S.A.* 110 12768–12773. 10.1073/pnas.1300318110 23781104 PMC3732935

